# Unexpected Oversolubility of CO_2_ Measured at Electrode–Electrolyte Interfaces

**DOI:** 10.1021/jacs.5c09712

**Published:** 2025-09-23

**Authors:** Zeke Coady, Samuel G. H. Brookes, Zhaohan Shen, Benjamin J. Rhodes, Grace Mapstone, Zhen Xu, Wei Yu, Hirotomo Nishihara, Christoph Schran, Angelos Michaelides, Alexander C. Forse

**Affiliations:** † Yusuf Hamied Department of Chemistry, 150385 University of Cambridge, Lensfield Road, Cambridge CB2 1EW, U.K.; ‡ Cavendish Laboratory, Department of Physics, 150385 University of Cambridge, J.J. Thomson Avenue, Cambridge CB3 0US, U.K.; § Institute of Multidisciplinary Research for Advanced Materials, Tohoku University, 2 Chome-1-1 Katahira, Aoba Ward, Sendai, Miyagi 980-0812, Japan; ∥ Department of Materials and Henry Royce Institute, University of Manchester, Oxford Rd, Manchester M13 9PL, U.K.; ⊥ Frontier Research Institute for Interdisciplinary Sciences, Tohoku University, Aoba-6-3 Aramaki, Aoba Ward, Sendai, Miyagi 980-0845, Japan; # Advanced Institute for Materials Research, Tohoku University, 2 Chome-1-1 Katahira, Aoba Ward, Sendai, Miyagi 980-8577, Japan

## Abstract

Enhancements in gas solubility in pore-confined liquids—termed oversolubility—can drastically influence gas separation and catalytic efficiency in confined environments; however, they remain poorly understood in electrochemical CO_2_ capture and reduction systems. While previous investigations of oversolubility have emphasized the importance of mesoporosity and incomplete pore saturation by the solvent, in this work, we report an unprecedented 30-fold oversolubility effect for CO_2_ in solely microporous activated carbons saturated with 1 M Na_2_SO_4(aq)_. The oversolubility effect occurs regardless of the activated carbon’s functional groups and level of disorder and is enhanced for smaller pore sizes. Oversolubility is quantified using solid-state ^13^C nuclear magnetic resonance spectroscopy (NMR), enabling differentiation between in-pore and ex-pore CO_2_ and HCO_3_^–^. Atomistic modeling of the system, based on a machine-learning model delivering first-principles accuracy, suggests that the effect is driven by an adsorption-like mechanism underpinned by favorable interactions between CO_2_ and the pore walls. Our findings demonstrate the unexpected importance of oversolubility for gas uptake in microporous, solvent-saturated carbon electrodes, an effect with direct relevance for improving electrochemical CO_2_ capture and conversion technologies.

## Graphical abstract

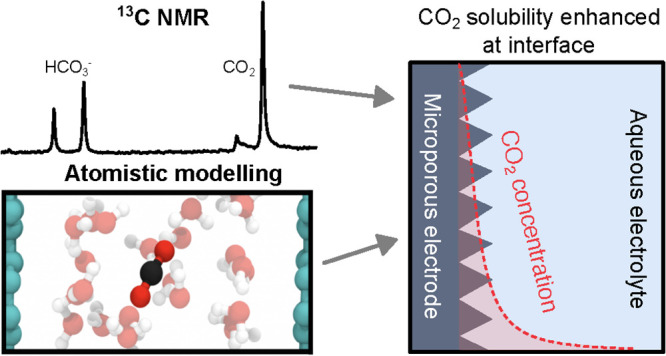

## Introduction

Confined gas–liquid–solid systems are becoming increasingly central to next-generation energy and environmental technologies. A particularly intriguing phenomenon in this context is oversolubility, which is the enhanced solubility of a gas in a solvent when confined in a porous material, as observed for a wide range of porous solids, solvents, and gases.^1–8^ Enhanced solubility is driven by changes in pore-confined solvent behavior compared to bulk solvent behavior.^9^

Oversolubility has been suggested as a method for enhancing CO_2_ capture and reduction methods, as well as other gas separation and catalysis processes.^9^ The incorporation of solvents into porous sorbent materials can result in a higher CO_2_ capacity than would be achieved in the solvent or sorbent alone due to an oversolubility effect.^10–18^ Oversolubility effects have also been observed to increase turnover number for CO_2_ utilization catalysts by affecting the local concentration of CO_2_.^1,3,6^ The potential for enhanced local concentration of CO_2_ is particularly promising for electrochemical CO_2_ capture and reduction processes. These processes often use porous electrodes in contact with a liquid electrolyte, creating conditions where an oversolubility effect may occur.^19–22^ Oversolubility therefore offers a tool to control CO_2_ concentration at electrode–electrolyte interfaces, and so enhance rates of CO_2_ capture or reduction reactions (Figure 1a).^23–26^

**Figure 1:**
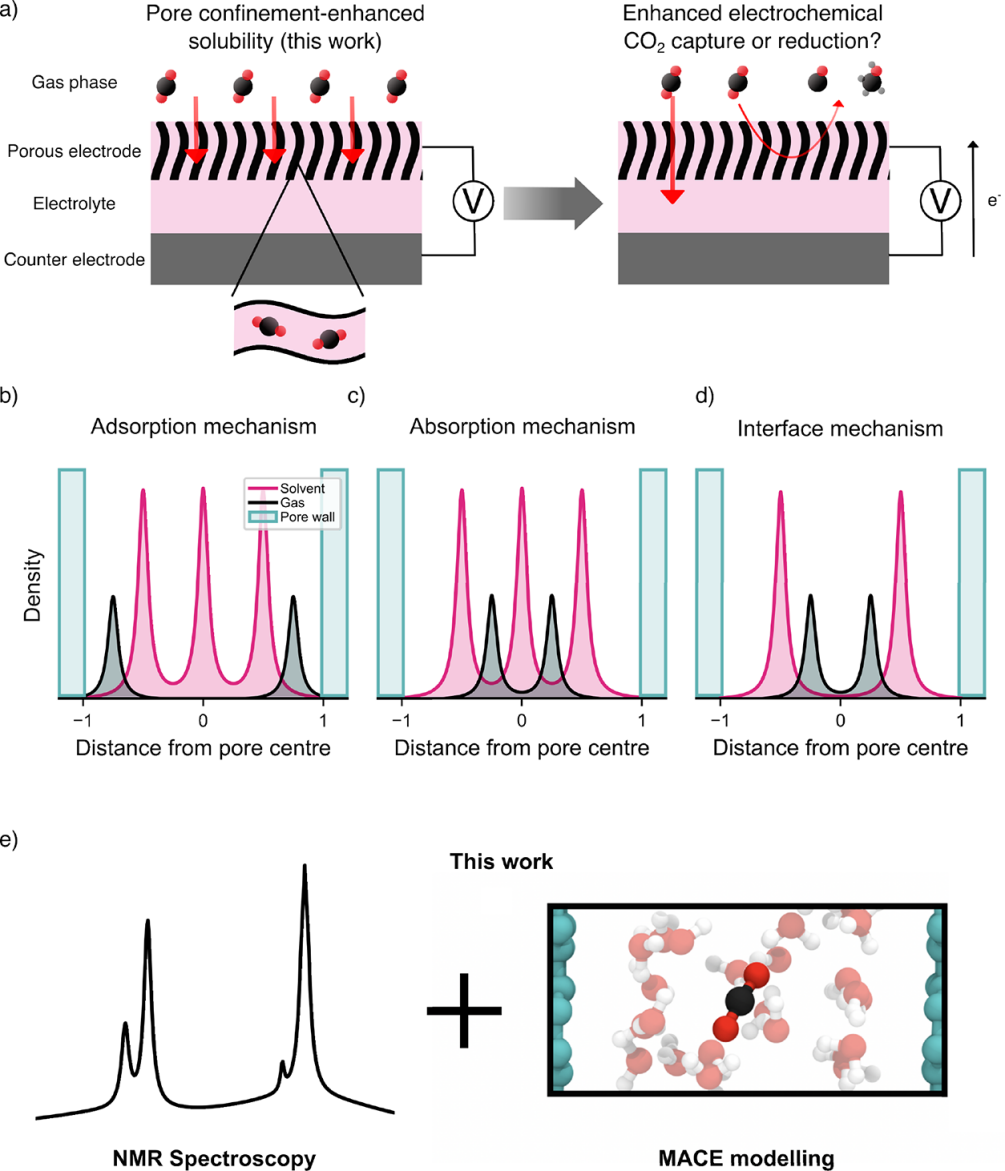
(a) CO_2_ solubility enhancement in porous electrodes (left) has the potential to enhance electrochemical CO_2_ capture and/or reduction (right). (b–d) Three mechanisms have been described in the literature to explain oversolubility, shown here schematically for idealized scenarios: (b) adsorption: enhanced concentrations of gas molecules reside at the pore–solvent interface; (c) absorption: enhanced concentrations of gas molecules reside between solvent layers; (d) interface: enhanced concentrations of gas molecules reside at the solvent–gas interface when the pores are incompletely saturated by the solvent. (e) In this work, NMR spectroscopy and machine-learning-driven atomistic modeling are applied to investigate CO_2_ oversolubility in soaked electrodes.

Understanding the role of oversolubility in electrochemical CO_2_ capture and reduction requires a firm understanding of the underlying chemistry. Previous literature reports suggest that oversolubility effects are most significant in mesoporous (pore size 2–50 nm) materials that are incompletely solvent-saturated.^2,27–29^ These are not strict requirements, as solubility enhancements have also been observed in solvent-saturated^29^ and microporous^18,30^ (pore size ≤2 nm) materials. These observations have been rationalized by developing three mechanisms of oversolubility based on computational studies of gas/liquid/porous solid systems.^2,9^ First, in the ‘adsorption’ mechanism, the adsorption of gas molecules onto the pore surface outcompetes interactions between the solvent and surface, and results in enhanced uptake of gas (Figure 1b). Second, in the ‘absorption’ mechanism, the gas is included in high-density layers between solvent layers which form inside pores (Figure 1c). Modeling suggests that the relative importance of the ‘adsorption’ and ‘absorption’ mechanisms is driven by the difference in strength between gas–solid and liquid–solid interactions.^2^ High surface area thus drives enhanced oversolubility,^31^ though this can be confounded by other effects such as pore size.^9,27^ Finally, the ‘interface’ mechanism (Figure 1d) occurs upon incomplete solvent-saturation, resulting in a high surface area gas/liquid interface where gas can accumulate. Oversolubility is therefore believed to be driven by gas inclusion at interfaces, due to a change in how the confined solvent phase behaves relative to the bulk solvent phase.

Despite the valuable insights obtained by previous experimental and computational work, literature investigations of oversolubility are insufficient for predicting its role in electrochemical CO_2_ capture and reduction processes. The porous electrodes used for electrochemical CO_2_ capture and reduction are solvent-saturated to allow for ion transport and usually contain micropores to enable a high surface area for CO_2_-electrode contact.^32^ No study exists examining the potential for oversolubility in materials that are both solvent-saturated and microporous. Accordingly, a number of open questions remain concerning the role of oversolubility in electrochemical CO_2_ capture and reduction. For instance, how does oversolubility influence the overall rate of uptake? How does the pore environment affect CO_2_ uptake? And how important are factors such as pore size, functional groups, and electrolyte ions in mediating oversolubility effects?

In this work, leveraging solid-state nuclear magnetic resonance (NMR) and state-of-the-art atomistic modeling techniques, we investigate the role of oversolubility in electrochemical CO_2_ capture and reduction processes. Recent developments have shown the efficacy of solid-state NMR spectroscopy for studying molecules and ions at electrode–electrolyte interfaces inside porous electrodes.^33–36^ Additionally, ^13^C NMR spectroscopy is a demonstrated method for understanding the uptake and speciation of CO_2_ in porous materials in the presence of water.^37–40^ Further, recent advances in atomistic modeling—driven by machine-learning based interatomic potentials—have enabled detailed simulations of complex systems and processes,^41–43^ including CO_2_-water interfaces^44^ and water under confinement.^45^ These methods can be used to run *ab initio*-level simulations at low computational costs, thereby allowing us to accurately sample large, complex systems over extended time scales.^45,46^ Combining these methods with NMR experiments is an established methodology for interpreting spectroscopic results.^47,48^

We find that an oversolubility effect significantly affects CO_2_ uptake in model electrochemical CO_2_ capture and reduction electrodes. ^13^C magic-angle-spinning (MAS) NMR spectroscopy measurements demonstrate that, in the presence of activated carbon, CO_2_ solubility in aqueous solvent is increased by up to a factor of 30. This is the first observation of oversolubility in a solvent-saturated microporous system. The solubility enhancement appears to increase in carbons with smaller pores among the three chosen activated carbons, and is retained in thermally annealed activated carbon. Atomistic modeling, carried out to provide an indirect assessment of oversolubility effects, suggests an adsorption-like mechanism is responsible for enhanced CO_2_ uptake, with energy calculations indicating a preferential location of CO_2_ over H_2_O at the pore walls. The application of combined NMR and molecular modeling techniques (Figure 1e) allows unique insights into the nature of CO_2_ solubility in solvent-saturated porous electrodes. This understanding and control of gas solubility in electrochemical CO_2_ capture and reduction provides a new tool to enhance the activity and turnover number in electrochemical CO_2_ capture and reduction systems.

## Results and Discussion

### Quantifying Oversolubility in Microporous Activated Carbons

^13^C NMR spectra were acquired for model activated carbon electrodes soaked in electrolyte and dosed with ^13^CO_2(g)_ (Figure 2a, top, and Figures S1–S3) to quantify CO_2_ uptake and HCO_3_^–^ formation and therefore potential oversolubility effects. The model electrode was based on a supercapacitive swing adsorption electrode,^19–21,49,50^ a simple electrochemical CO_2_ capture system. A small peak for gaseous or aqueous CO_2_ was observed at 125 ppm, in line with literature.^51–55^ The peak at approximately 120 ppm was assigned to CO_2_ inside the carbon pores, with the change in chemical shift (δ) due to a nucleus-independent chemical shift effect.^33,35,56^ This was confirmed by comparison with the physisorbed CO_2_ peak observed in ^13^C NMR spectra of the equivalent dry activated carbon samples dosed with ^13^CO_2(g)_ (Figures S4–S6). As the spectra were obtained under quantitative conditions, integration of the in-pore CO_2_ peak allows quantification of CO_2_ uptake by the combined electrolyte–electrode system, and therefore measurement of CO_2_ molality (*b*_CO_2__). Integration was correlated with molar uptake of CO_2_ through calibration to gas sorption experiments (Figure S7). Peaks at approximately 160 and 155 ppm were also observed in the wetted samples, indicating formation of HCO_3_^–^ in the sample in both the in-pore and ex-pore environments.^52,57,58^ These were similarly integrated to measure HCO_3_^–^ molality (*b*_HCO_3_^–^_). Measurement of molality rather than molarity avoids issues caused by possible changes in solvent density under confinement.^59^ Summing *b*_CO_2__ and *b*_HCO_3_^–^_ gave the total molality of dissolved gas (*b*). The solubility enhancement (SE) can then be calculated as in eq 1 by dividing *b* by the literature solubility of CO_2_ in bulk solvent (*b*_bulk_).^60,61^

$$\mathrm{SE}=\frac{b}{b_{\mathrm{bulk}}}=\frac{b_{{\mathrm{CO}}_{2}}+b_{{\mathrm{HCO}}_{3}^{-}}}{b_{\mathrm{bulk}}}$$ 1

**Figure 2:**
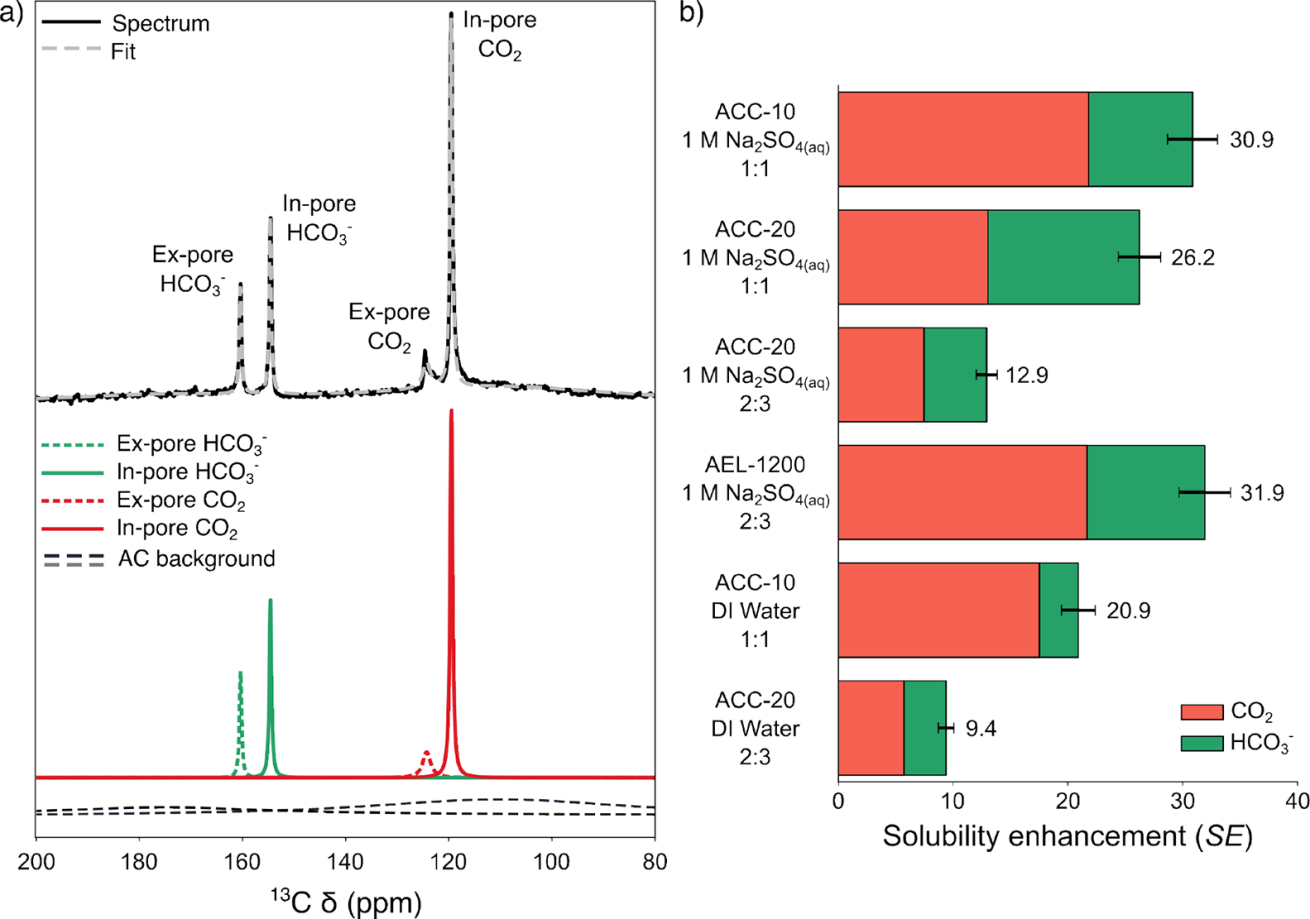
Quantitative ^13^C NMR spectra show oversolubility of CO_2_ in porous carbons. (a) ^13^C NMR spectrum (9.4 T, 5 kHz MAS) of ^13^CO_2(g)_–dosed ACC-20/1 M Na_2_SO_4(aq)_ (2:3 mass/volume ratio) (top, black), fitted spectrum (top, gray), and individual deconvoluted peaks of the fit showing the four sharp CO_2_-derived peaks and the two broad ACC-20 peaks (bottom). (b) Solubility enhancement (*SE*) for CO_2_ in activated carbon/solvent samples based on quantitative NMR measurements. Enhancements are divided into CO_2_ solubility (red) and HCO_3_^–^ formation (green). Enhancements are calculated relative to literature values for CO_2_ solubility in bulk solvents.^60,61^

Additionally, a broad background signal is observed to overlap the sharp CO_2_-derived peaks. This was assigned to ^13^C nuclei in the activated carbon, based on ^13^C NMR measurements of the undosed activated carbon (Figures S8 and S9). This can be modeled in the spectrum deconvolution by addition of two broad Lorentzian peaks (Figure 2a, bottom). The Supporting Information contains further discussion of NMR spectra assignment and calculations, as well as a full list of calculated molalities and solubilities (Table S2).

We observed significant CO_2_ oversolubility in the studied model systems, driven by both increased CO_2_ dissolution and significant formation of HCO_3_^–^. Figure 2b shows the NMR-derived SE for several different activated carbon/aqueous solvent systems, with up to 30-fold enhancements observed across the systems examined in this work. SE can be divided into contributions from CO_2_ dissolution (SE_CO_2__, red) and from HCO_3_^–^ formation (SE_HCO_3_^–^_, green). Considering only CO_2_ dissolution, a 5- to 20-fold enhancement was still observed depending on the choice of activated carbon and solvent. This is comparable to literature measurements of oversolubility in other systems, which also are on the order of 5– to 20-fold in most cases.^1–9^ The ability to distinguish between dissolution of CO_2_ and formation of HCO_3_^–^ demonstrates a key advantage of NMR spectroscopy methods for probing oversolubility.

### Effects of Different Activated Carbons and Solvents

Solubility enhancements were measured for three different activated carbons (ACC-10, ACC-20, and AEL-1200) to understand the role of the activated carbon structure in oversolubility (Figure 2b). The difference between ACC-10 and ACC-20, two activated carbon cloths which have both been previously applied for capacitive CO_2_ capture,^21^ bear particular discussion. The two cloths are chemically and structurally similar, with ACC-10 having a smaller average pore size, total surface area, and pore volume than ACC-20, and similar surface area-to-volume ratio (Table S3).^21,33^ ACC-10 showed a higher SE than ACC-20 (30.9× compared to 26.2× for a 1:1 mg:μL mixture). This is surprising, as the ACC-10 sample has both a lower surface area and a lower pore volume than ACC-20 does, relative to the amount of water in the system. Indeed, ^1^H NMR experiments indicate that less of the solvent in the ACC-10 sample resides inside the activated carbon’s pores compared to the ACC-20 sample (Figures S10–S12). This suggests that smaller pore size could be a driving factor for the solubility enhancement.

In addition, the ACC-20 sample contains more HCO_3_^–^, relative to CO_2_, than the ACC-10 sample (Figure 2b). Both ACC carbons have basic surfaces in 1 M Na_2_SO_4(aq)_, based on measurement of the apparent point of zero charge using the pH-drift^62^ procedure (Figure S13). As ACC-20 has a higher surface area than ACC-10, it is proposed that the electrolyte solution in this system is more basic, resulting in a swing in the CO_2_–HCO_3_^–^ equilibrium inside the pores. To test this, CO_2_ oversolubility was measured in a 2:3 mg:μL mixture, which possesses a similar carbon surface area:water volume ratio to the ACC-10 1:1 mg:μL sample. The CO_2_:HCO_3_^–^ equilibrium in the 2:3 mg:μL sample shifts toward CO_2_ compared to the 1:1 mg:μL sample (Figure 2b), as predicted. In addition, SE was reduced in the 2:3 sample compared to the 1:1 sample, as while the amount of CO_2_ present is similar in each sample, the mass of water present is much higher in the 2:3 sample.

To investigate the role of functional groups and disorder in the activated carbon, CO_2_ solubility was also investigated in the activated carbon AEL-1200, which was annealed at 1200 °C to remove functional groups. To confirm the loss of functional groups, temperature-programmed desorption (TPD) analysis up to 1800 °C was carried out,^63^ which indicated a lower oxygen content compared to the ACC carbons, as well as larger graphene-like domains (Table S4). Since SE in AEL-1200/1 M Na_2_SO_4_(aq) was higher than for either of the ACC carbons (Figure 2b), CO_2_ oversolubility is therefore not driven by the involvement of functional groups. This further supports that oversolubility in these activated carbon systems is driven by interactions between CO_2_ and the graphene-like pore walls of activated carbon when under confinement, rather than between CO_2_ and functional groups or defect sites.

The oversolubility effect was also observed to be general for aqueous solvents. 1 M Na_2_SO_4(aq)_ was initially used as the solvent to replicate the ionic electrolyte used in electrochemical CO_2_ capture and reduction systems. The presence of additional ions in the system raised the question of whether this is a general enhancement in aqueous systems. SE for CO_2_ was therefore also measured for ACC-10 and ACC-20/deionized water systems (Figure 2b), and a significant solubility enhancement was still observed in the absence of Na^+^ and SO_4_^2–^. Deionized water systems showed higher overall CO_2_ solubility than the equivalent 1 M Na_2_SO_4(aq)_ systems, but the overall order of the enhancement relative to CO_2_ solubility in the bulk system was lower, resulting in lower SE values.

NMR spectroscopy also enables direct quantification of the solubility enhancement for the solvent contained inside the pores of the porous sorbent. Since separate in-pore and ex-pore resonances are observed for solvent as well as CO_2_ and HCO_3_^–^ (Figure 3a), in-pore molalities (*b* ^in‑pore^) and in-pore solubility enhancements ((SE^in‑pore^)) can be calculated for all systems (Figure 3b and eq 2).

$${\mathrm{SE}}^{\mathrm{in‐pore}}=\frac{b^{\mathrm{in‐pore}}}{b_{\mathrm{bulk}}}=\frac{b_{{\mathrm{CO}}_{2}}^{\mathrm{in‐pore}}+b_{{\mathrm{HCO}}_{3}^{-}}^{\mathrm{in‐pore}}}{b_{\mathrm{bulk}}}$$ 2

Notably, SE^in‑pore^ calculated for the 1:1 and 2:3 systems for ACC-20 are within error of each other, supporting our suggestion that the in-pore solvent is the critical species in the system. SE^in‑pore^ is therefore proposed as a measure of oversolubility for solvent-saturated systems which is agnostic of the added solvent volume, and depends only on the choice of porous solid and solvent. SE^in‑pore^ is also observed to be higher for ACC-10 than ACC-20, supporting the proposed dependence on pore size, and higher for AEL-1200 than ACC-20, suggesting the significance of ordered graphene-like domains.

### Atomistic Simulations to Investigate the Mechanism of Oversolubility

The observation of an oversolubility effect is rare for microporous materials.^2,9^ To better understand the mechanism underpinning CO_2_ uptake, we carried out a series of atomistic simulations aimed at modeling idealized pore environments. These simulations were performed to provide an indirect assessment of oversolubility and to help interpret some of the spectroscopic measurements discussed above. The simulations were enabled by MACE potentials^64^ trained on a representative sample of structures labeled with energies and forces calculated at the revPBE-D3 level.^65–67^ The objectives of this modeling were threefold: to understand the underlying mechanism for CO_2_ oversolubility; to provide a quantitative rationale for observing this mechanism; and to elucidate the microscopic details driving the process. Results and analyses of this work are provided in the following section, with full computational details and setups reserved for the Supporting Information.

To gauge the underlying mechanism for solute uptake in microporous carbon, we performed free molecular dynamics simulations modeling the behavior of CO_2_ and HCO_3_^–^ in water-saturated pore environments. Simulations were carried out for pore widths of 7, 12, and 15 Å, corresponding to the system-relaxed separations that support layered water structures. These separations also allowed us to fully sample the pore size distributions of our chosen microporous carbons (Figure S14). In each setup, a single CO_2_ or HCO_3_^–^ molecule/ion was allowed to move freely through the pore environment; this gave the closest possible CO_2_:H_2_O or HCO_3_^–^:H_2_O mole fraction to the experimentally measured values (Table S5). Simulations were performed over several nanoseconds, allowing for fully converged statistics of the in-pore properties.

The resulting density profiles, shown separately for H_2_O, CO_2_, and HCO_3_^–^, are given in Figure 4a. These profiles reveal the important role of the pore-water interface. In all systems, the pore geometry results in the formation of layered water structures (pink).^68^ Analysis of the CO_2_ and HCO_3_^–^ profiles reveals that both species preferentially locate within the H_2_O layers, albeit with distinct positional preferences. Across each pore width, CO_2_ (black) exhibits a strong affinity for the water layer in contact with the pore wall. In contrast, HCO_3_^–^ (purple) appears less constrained and is less likely to locate at the pore-water contact layer. This is particularly noticeable for larger pore widths. Although reactivity in these systems was possible, no exchange between CO_2_ and HCO_3_^–^ was observed over the studied time scales.

**Figure 3:**
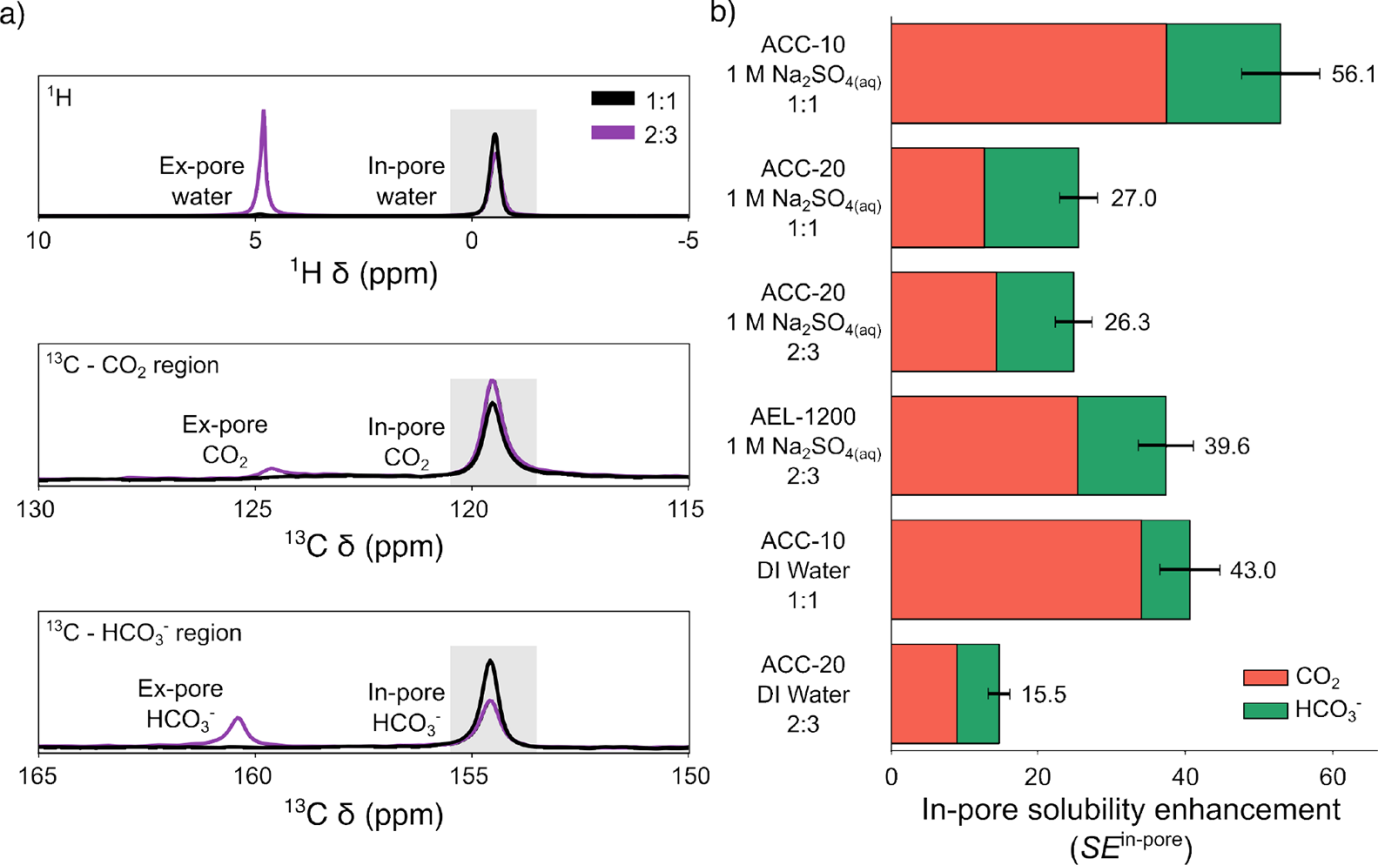
NMR spectroscopy allows the measurement of SE^in‑pore^ using only in-pore water. (a) Ex-pore and in-pore NMR peaks (9.4 T, 5 kHz MAS) of water, CO_2_, and HCO_3_^–^ for ACC-20/1 M Na_2_SO_4(aq)_ in a 1:1 (black) and 2:3 (purple) mass/volume ratio. Gray boxes show the in-pore regions used to calculate solubility enhancements for the in-pore material only. (b) In-pore solubility enhancement (SE^in‑pore^) in activated carbon/solvent samples. SE^in‑pore^ is calculated relative to the in-pore water in the system and should be agnostic to the total solvent volume.

The profiles shown in Figure 4a support an adsorption-like mechanism of oversolubility for CO_2_ (see Figure 1b). Uptake appears to be driven by a preferential adsorption of CO_2_ at the pore walls, evidenced by the pronounced peaks in CO_2_ density adjacent to the graphene distributions. To understand and quantify this behavior, we measured the adsorption energies of CO_2_ and H_2_O under gaseous and pore-saturated conditions. In one approach, we computed single-molecule interaction energies of gaseous CO_2_ and H_2_O molecules interacting with isolated pore walls (Figure 4b). In a complementary approach, enhanced sampling simulations of pore-saturated systems (30 Å width) were performed to obtain free energies as a function of the distance from the pore wall (Figure 4c). Together, these two analyses allowed us to gauge the relative affinities of CO_2_ and H_2_O for the pore wall both on a per-molecule basis and also accounting for solvation effects from water.

Both sets of results confirm the preferential adsorption of CO_2_ over H_2_O at the pore wall. In the gas phase, CO_2_ adsorption is more favorable by 0.34 kcal/mol, while under pore-saturated conditions, CO_2_’s free energy of adsorption is 0.22 kcal/mol lower (more stable) than that of H_2_O. Likely, these enhanced stabilities arise from favorable dispersion interactions occurring between hydrophobic CO_2_ and the basal graphene planes.^69^ In addition, sequestering CO_2_ at the pore-water interface minimizes disruption to the overall hydrogen bonding network, with water molecules located at the pore wall forming fewer hydrogen bonds on average compared to bulk water (see Figure S18). The free energy plot obtained for CO_2_ is consistent with the density profiles shown in Figure 4a, with a free energy minimum located adjacent to carbon and an unstable regime (+1 kcal/mol) located 5–8 Å from the pore wall. We note that, for this large pore environment (30 Å, Figure 4c), we recover bulk-like water toward the center of the idealized pore slit (i.e., toward 15 Å). In this way, the analysis of Figure 4c further evidences the preference of CO_2_ to locate in pore environments versus in bulk solution. The favorability of the CO_2_-pore wall interaction relative to the H_2_O-pore wall interaction predicts that the adsorption mechanism of oversolubility will dominate.^2,9^ The unstable regime located 5–8 Å from the pore wall additionally indicates that the absorption mechanism is unfavorable.

Having confirmed the preferential adsorption of CO_2_ at the pore wall over H_2_O, we then investigated the manner in which CO_2_ is adsorbed. In Figure 4d, orientational distributions for CO_2_ and H_2_O molecules residing at the contact layer of the pore-water interface are shown. Orientations are measured by the angle between the *z* vector normal to the pore wall and either the C–O bond vector of CO_2_ or the dipole vector of H_2_O. Snapshots of the contact layer parallel and perpendicular to the *z* vector are shown in Figure 4e.

Looking at Figure 4d, we observe a peak in the distribution for CO_2_ at 90°, suggesting that CO_2_ absorbs mainly parallel to the pore wall. In contrast, H_2_O adsorbs across a broad distribution of orientations with a loose preference for two specific geometries: with dipoles pointing slightly toward the bulk (60°, most favored); and with dipoles pointing slightly toward the pore walls (105°, less favored). These observations are consistent with previous modeling of graphene–water interfaces.^70^ The parallel adsorption of CO_2_ likely enables more favorable interactions with the pore walls and facilitates the diffusion of CO_2_ into narrow pore environments. Similar mechanisms have been observed to enhance CO_2_ capture in dry activated carbon pores^71^ and to enhance selectivity for CO_2_ over H_2_O in metal–organic framework pores.^72^ In contrast, water’s rough bimodal adsorption could present an obstacle to narrow-pore diffusion, precluding the uptake of H_2_O into more confined environments. This may help explain the stronger oversolubility effect observed for ACC-10 compared to ACC-20, given the smaller average pore size of the former.

**Figure 4:**
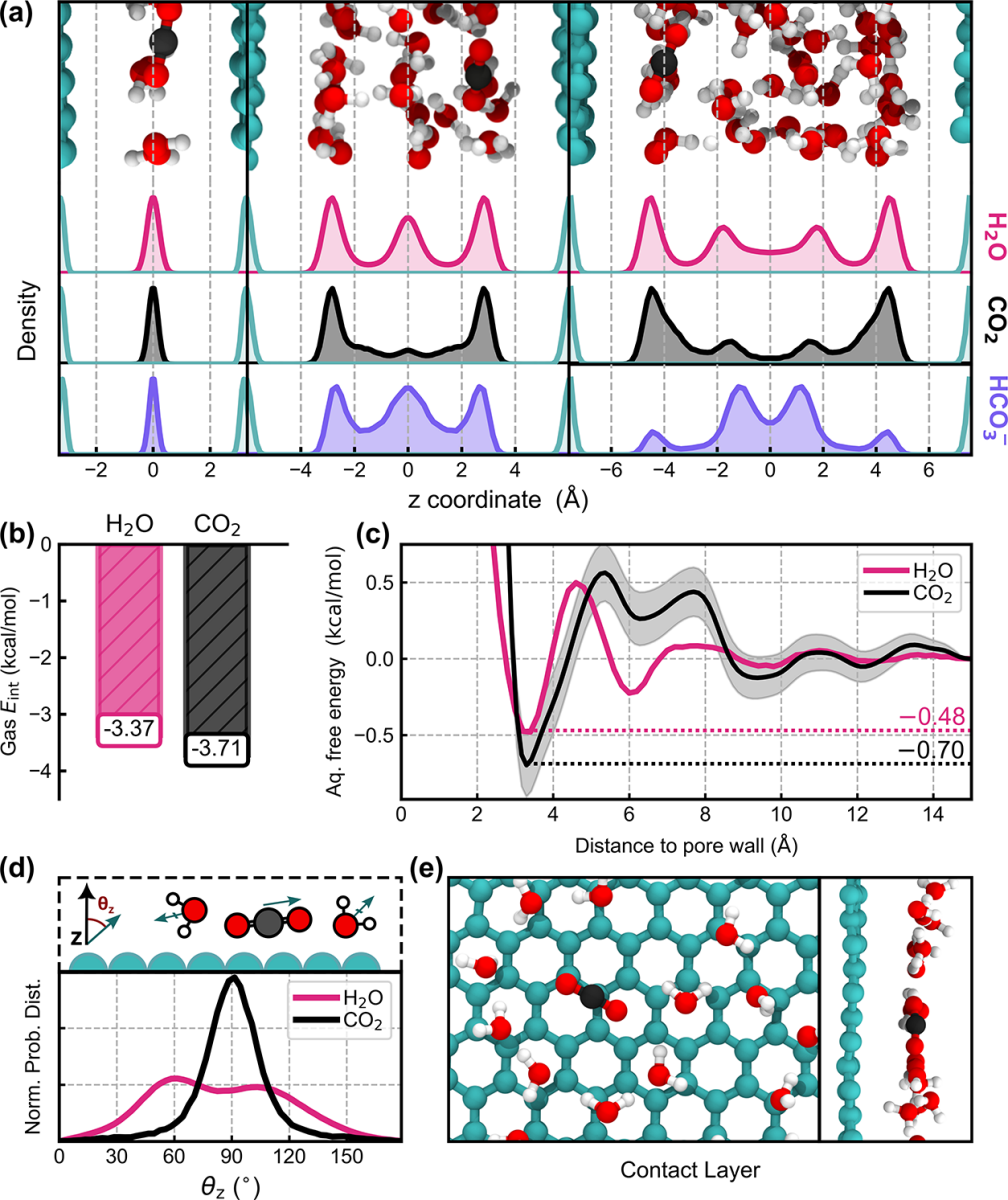
Atomistic modeling suggests CO_2_ uptake is driven by the adsorption mechanism. (a) Density profiles for water (pink), CO_2_ (black), and HCO_3_^–^ (purple), plotted as a function of the distance from the system center of mass. Profiles are obtained for 7 Å (left), 12 Å (center), and 15 Å (right) pores. (b) Interaction energies for isolated gaseous CO_2_ and H_2_O molecules interacting with a graphene-like pore wall. (c) Free energy profiles of CO_2_ and H_2_O shown as a function of the distance from the pore wall under pore-saturated conditions (30 Å confinement). (d) Orientational distributions of the CO_2_ bond vector and H_2_O dipole vectors obtained for molecules at the pore–water contact layer. (e) Visualization of the contact layer of the pore–water interface.

Overall, the atomistic modeling of idealized slit pores suggests the following: the uptake of CO_2_ in water-saturated microporous carbon is driven by the adsorption mechanism; this mechanism arises due to a preferential adsorption of CO_2_ over H_2_O at the pore-water interface; and CO_2_ will adsorb parallel to the pore walls, potentially facilitating further uptake enhancements under narrow pore environments. While the pore-slit model employed here may be relatively simple compared to the actual activated carbon pore environment,^73^ the higher SE observed in AEL-1200 compared to ACC-10 and ACC-20 supports the significance of simple, graphene-like domains for modeling this uptake effect. Accordingly, we can have confidence in the conclusions drawn from this analysis and their applicability to more complex pore environments.

## Conclusions

In conclusion, this work demonstrated a novel CO_2_ oversolubility in solvent-saturated microporous carbon electrodes, identifying a new route for controlling electrochemical CO_2_ capture and reduction processes. ^13^C NMR spectroscopy methods allowed quantitative determination of CO_2_ solubility and insight into chemical and physical speciation. Quantification of the in-pore water enabled calculation of the volume-agnostic SE^in‑pore^ in the studied carbons. Observation of this oversolubility across multiple activated carbons and solvents indicated that CO_2_ uptake was a general effect in solvent-saturated microporous carbons. Oversolubility was enhanced in pores narrower than 1 nm and in highly ordered pores.

Machine-learning driven atomistic modeling of pore environments revealed that CO_2_ oversolubility was driven by the adsorption mechanism. Density profiles revealed a propensity for CO_2_ to adsorb at the pore-water interface, a phenomenon attributed to favorable interactions between CO_2_ and the pore walls. Adsorption of CO_2_ parallel to the surface likely facilitates uptake in smaller, more constrained pore environments, in line with experimental results. This suggests that oversolubility can be controlled through the deliberate design of porous carbon electrodes, including tuning pore size, functional group content, and graphene domain size.

Extending our combined NMR-modeling approach to disordered and defective carbon structures will be critical to validate this hypothesis and refine our understanding of microporous activated carbon. Work is underway to combine our modeling approach with more realistic carbon structures^73^ as well as structures incorporating defect sites. Future research will also examine CO_2_ speciation through additional NMR experiments examining the effect of carbon structure on the in-pore chemical shift,^33^ on diffusion behavior,^18^ and on chemical and physical exchange at the interface. This research will provide additional insight into the relationship between carbon structure and oversolubility. Taking advantage of the relationship between carbon structure and oversolubility will allow future electrochemical CO_2_ capture and reduction studies to control CO_2_ solubility at the electrode–electrolyte interface. Increasing the local concentration of CO_2_ could accelerate capture and conversion kinetics, though further insight into the balance between stabilization and reactivity will be necessary. Detailed spectroscopic studies of CO_2_ speciation, especially in nanoconfined aqueous environments, will be instrumental in this regard. More broadly, the concept of confinement-driven oversolubility should be considered in the design of electrochemical systems reliant on gas–liquid–solid interactions, including Li–CO_2_, Zn-CO_2_, Li–air, and Zn–air batteries.^74,75^

## Supplementary Material



## Data Availability

All raw experimental data files are available in the Cambridge Research Repository, Apollo, with the DOI identifier: 10.17863/CAM.119248. All data required to reproduce the computational findings of this work can be found in the following repository: https://github.com/water-ice-group/oversolubility.

## Notes

Materials and methods, calculated solubility and solubility enhancement values, gas sorption results, TPD measurements, comparisons of mole fractions in experiments and simulations, NMR spectra, NMR integration calibration curves, point of zero charge measurements, activated carbon pore size distributions, model validation tests, and additional computational pore-slit analysis (PDF)
