# Supplementary material for: Unexpected Oversolubility of CO2 Measured at Electrode–Electrolyte Interfaces
Source: J Am Chem Soc. 2025 Sep 23;147(40):36310–9. doi: 10.1021/jacs.5c09712 (PMC12512178; doi:10.1021/jacs.5c09712)
Supplement: Supplementary file 1 [file ja5c09712_si_001.pdf]

Supplementary Information - Unexpected oversolubility of CO_2 measured at electrode-electrolyte interfaces

Zeke Coady,[†] Samuel G. H. Brookes,[†,‡] Zhaohan Shen,[¶] Benjamin J. Rhodes,[†] Grace Mapstone,[†] Zhen Xu,[§] Wei Yu,[‖,⊥] Hirotomo Nishihara,[¶,⊥] Christoph Schran,[*,#] Angelos Michaelides,[*,†] and Alexander C. Forse[*,†]

†*Yusuf Hamied Department of Chemistry, University of Cambridge, Lensfield Road, Cambridge CB2 1EW, United Kingdom*

‡*Cavendish Laboratory, Department of Physics, University of Cambridge, JJ Thomson Avenue, Cambridge CB3 0US,, United Kingdom*

¶*Institute of Multidisciplinary Research for Advanced Materials, Tohoku University, 2 Chome-1-1 Katahira, Aoba Ward, Sendai, Miyagi 980-0812, Japan*

§*Department of Materials and Henry Royce Institute, University of Manchester, Oxford Rd, Manchester M13 9PL, United Kingdom*

‖*Frontier Research Institute for Interdisciplinary Sciences, Tohoku University, Aoba-6-3 Aramaki, Aoba Ward, Sendai, Miyagi 980-0845, Japan*

⊥*Advanced Institute for Materials Research, Tohoku University, 2 Chome-1-1 Katahira, Aoba Ward, Sendai, Miyagi 980-8577, Japan*

#*Cavendish Laboratory, Department of Physics, University of Cambridge, JJ Thomson Avenue, Cambridge CB3 0HE, United Kingdom*

E-mail: cs2121@cam.ac.uk; am452@cam.ac.uk; acf50@cam.ac.uk

Materials and methods

Materials

Activated carbon cloths ACC-5092-10 (ACC-10) and ACC-5092-20 (ACC-20) were purchased from Kynol. Activated carbon cloths were washed with approximately 500 mL of deionized water (per 5 g of carbon) and dried in a vacuum oven at 90ºC for 24 hours before storage under atmospheric conditions.

AEL-1200 was prepared from the activated carbon PowerSorb EL-104, obtained from Jacobi (Jacobi Carbons Group), by annealing at 1200ºC for 5 hours under Ar gas, according to the method described in Liu et al.[1] AEL-1200 was then made into a free-standing carbon film by mixing 95 wt % activated carbon with 5 wt % polytetrafluoroethylene binder (Sigma-Aldrich, 60 wt % dispersion in water) in ethanol. The film was manually rolled to 0.25 mm thickness and dried in a vacuum oven at 90ºC for 24 hours to remove residual water and ethanol before storage under atmospheric conditions.

Sodium sulfate (anhydrous, ≥99%) salt was purchased from Fisher Scientific and then made into a 1.00 M aqueous solution with deionized water.

^{13}C-enriched CO_2 gas was purchased from Cambridge Isotope Laboratories, with 98 at.% ^{13}C.

Preparation and $^{13}CO_2$ dosing of solid-state NMR samples

Activated carbon/solvent samples were prepared to mimic conditions observed in supercapacitive swing adsorption, a notable electrochemical CO_2 capture technique.[2,3]

For each activated carbon/solvent sample, the activated carbon (either cloth or film) was cut into small pieces, then an approximately 10 mg sample was weighed and placed in a vial. Solvent (either 1 M $Na_2SO_{4(aq)}$ or deionized water) was then added to achieve either a 1:1 or 2:3 mass/volume ratio between the activated carbon and solvent. The vial was sealed and left for 5 minutes to allow solvent to saturate the sample, then packed into a 3.2 mm or 4

mm rotor as quickly as possible to minimize evaporation of the solvent. Samples and vessels were weighed before and after addition of solvent and packing to quantify any loss of solvent due to evaporation.

Each sample was briefly evacuated for 1 minute under a static vacuum in a home-built gas manifold, as described previously.[4] Samples were then dosed with $^{13}CO_{2(g)}$ at room temperature at an initial atmosphere of 0.7 bar for 30 minutes, before sealing the rotors inside the gas manifold with a mechanical plunger.

Solid-state NMR Spectroscopy Experiments

NMR spectroscopy experiments were carried out with a Bruker Avance Neo spectrometer in a Bruker 3.2 mm HXY triple resonance probe or a Bruker 4 mm HX double resonance probe. Measurements were carried out at a magnetic field strength of 9.4 T, corresponding to a ^{1}H Larmor frequency of 400.1 MHz. All spectra were acquired with a 90º pulse-acquire sequence at an MAS speed of 5 kHz, at which speed we observe resolved spectra while avoiding excess frictional heating or centrifugation.[5,6] The 90º pulse length was optimized for each sample. Recycle delays were set to $> 5T_1$ for the CO_2-derived peaks for each sample to ensure measurements were quantitative, based on measurements of T_1 through inversion recovery experiments. Measurements were not quantitative for the activated carbon background peaks. ^{13}C and ^{1}H NMR spectra were referenced relative to the ^{13}C CH resonance of adamantane at 37.78 ppm as a secondary reference, using $\Xi(^{13}C) = 25.145972\%$ to reference the ^{1}H spectra.[7–9]

NMR spectra were deconvoluted and fitted using ssNake.[10] The fitting of each spectrum was repeated at least 3 times. For each fitting, different initial peak shapes were applied, then varied freely to achieve the optimal fitting.

^{13}C spectrum peak assignment

Up to four sharp peaks were observed in activated carbon/solvent samples after dosing with $^{13}CO_{2(g)}$. Peak shifts varied slightly depending on the choice of activated carbon and solvent.

Observation of paired peaks, separated by approximately 5 ppm, was consistent with previous observations of ex-pore and in-pore environments in activated carbons, with the magnitude of the separation depending on the structure of the activated carbon.[1,5,6,11,12]

CO_2 is observed at 124-126 ppm in literature in both gaseous and aqueous phases.[13–17] The ^{13}C NMR spectrum of $^{13}CO_2$-dosed dry ACC-20 (see Fig. S4) showed a strong resonance at 119 ppm resulting from physisorbed in-pore CO_2. No ex-pore CO_2 was observed in Fig. S4 due to the low intergranular space in the carbon,[2] low density of gas molecules, the peak-broadening effects of exchange,[18] and overlap with background activated carbon peaks (discussed below). Therefore, the two right-most environments in the activated carbon/solvent samples, observed at 124-125 and 117-120 ppm, were assigned to ex-pore and in-pore CO_2 respectively. Determining whether the ex-pore peak is aqueous or gaseous is difficult due to the small intensity of the peak in most spectra. In some ^{13}C NMR spectra, the ex-pore CO_2 peak's shape suggests the potential for a two-peak fitting, suggesting that both aqueous and gaseous CO_2 are present, but deconvolution was not carried out due to the potential for overfitting on such a small signal. Measurement of T_1 could also potentially be used to identify which phases are present,[15] since aqueous and gaseous CO_2 would exhibit much different relaxation behavior, but due to the low signal, potential effects of exchange, and significant background from the activated carbon, this analysis was not possible.

The leftmost environment, at approximately 160 ppm was assigned to ex-pore bicarbonate based on literature measurements.[14,19,20] While bicarbonate and carbonate are expected to be observed in one environment by NMR due to rapid exchange, we can estimate that this environment is effectively 100% bicarbonate, based on literature measurements showing the relationship between chemical shift and the bicarbonate-carbonate equilibrium.[20] The peak at 150-155 ppm was therefore assigned to in-pore bicarbonate.

The broad background peaks were assigned to activated carbon, based on ^{13}C NMR spectra of activated carbon and activated carbon/solvent samples without $^{13}CO_{2(g)}$ dosing (Figs. S8 to S9). A peak at approximately 120 ppm was observed in all activated carbon samples and was assigned to the aromatic carbon environments which make up the majority of carbon atoms in an activated carbon material. A peak at approximately 170 ppm was observed in some carbons and was assigned to a spinning sideband of the 120 ppm peak. We also cannot rule out contribution from C=O environments, given the known presence of oxygen in the activated carbon samples (Tab. S4). A peak at approximately 70 ppm was also observed in some carbons and was assigned to a spinning sideband of the 120 ppm peak. This peak did not contribute to the spectral region considered for NMR quantification of CO_2 uptake, and was not included in deconvolution. These assignments align with previous results from ^{1}H-^{13}C cross-polarization measurements of carbon materials.[21–23]

Measurements of solubility enhancement

Calibration of NMR integration to CO_2 uptake

To calibrate the relationship between NMR integration and CO_2 uptake, ACC-20 samples without solvent were prepared and packed in 3.2 mm rotors. These samples were evacuated for 1 hour to remove adsorbed water, then were dosed with $^{13}CO_{2(g)}$ in the same manner as the wet samples at a range of initial atmospheres between 0.4 and 0.8 bar for 30 minutes before sealing the rotors inside the gas manifold with a mechanical plunger.

Uptake of adsorbed CO_2 in each sample was measured through integration of the corresponding NMR peak for in-pore CO_2. A calibration constant relating this integration to moles of CO_2 was calculated by comparing to the volumetric CO_2 sorption isotherm for ACC-20 (see Fig. S7).

Calibration on $^{13}CO_{2(g)}$-dosed ACC-20 was performed to account for potential effects of the skin depth effect, which may attenuate NMR signals in the conductive activated carbon samples.[24–26]

Quantification of CO_2 uptake in activated carbon/solvent samples

Moles of in-pore CO_2 ($moles_{CO_2}^{\text{in-pore}}$), of in-pore HCO_3^- ($moles_{HCO_3^-}^{\text{in-pore}}$), and of ex-pore HCO_3^- ($moles_{HCO_3^-}^{\text{ex-pore}}$) were calculated from integration of the corresponding NMR peaks, using the calculated calibration constant.

To estimate error, three $^{13}CO_{2(g)}$-dosed 2:3 m/v ACC-20/1 M $Na_2SO_{4(aq)}$ were prepared, the ^{13}C NMR spectra recorded, and $moles_{CO_2}$ and $moles_{HCO_3^-}$ were calculated for each. The standard deviation divided by the mean was 5.6% for $moles_{CO_2}$ and 7.2% for $moles_{HCO_3^-}$. Variation in m_{water}, based on the inherent error of our mass balance, was negligible in comparison and has been disregarded. The 7.2% standard deviation is given for error bars in our plots for all samples to provide an estimate of potential variance in signal from $^{13}CO_2$-dosed activated carbon/solvent samples.

Background signal from activated carbon was removed by subtraction of the spectrum of undosed carbon/electrolyte prior to integration. This approach was used as otherwise deconvolution (performed in ssNake) gave inconsistent values for peak integration upon repeating fitting procedures, resulting in very high errors in final calculations.

Calculation of molality

Total dissolution of CO_2 (b) for activated carbon/solvent systems was calculated according to Eq. (1):

$$b = \frac{moles_{CO_2}^{\text{in-pore}} + moles_{HCO_3^-}^{\text{in-pore}} + moles_{HCO_3^-}^{\text{ex-pore}}}{m_{\text{water}}} \tag{1}$$

Ex-pore CO_2 was not included in the calculation of due to the possibility of contribution from $CO_{2(g)}$. m_{water} is the mass of water inside the NMR rotor, and was calculated using Eq. (2).

$$m_{\text{water}} = m_{\text{water, initial}} \times \frac{m_{\text{all, rotor}}}{m_{\text{all, initial}}} \tag{2}$$

$m_{\text{all, initial}}$ is the total activated carbon/solvent mass used in sample preparation, while $m_{\text{all, rotor}}$ is the mass of the system after packing into the rotor. $m_{\text{water, initial}}$, the mass of water in the initial sample, was calculated from the mass of solvent added to the initial sample ($m_{solvent,initial}$) and the mass fraction of water in the solvent (w_{water}) as in Eq. (3), accounting for water loss due to evaporation during sample preparation (m_{evap}); this was normally negligible.

$$m_{\text{water, initial}} = m_{\text{solvent, initial}} \times w_{\text{water}} - m_{\text{evap}} \tag{3}$$

Species-specific molality, such as for CO_2 only (b_{CO_2}), was calculated by modifying Eq. (1) to only include certain species, as in Eq. (4):

$$b_{CO_2} = \frac{moles_{CO_2}^{\text{in-pore}}}{m_{\text{water}}} \tag{4}$$

In-pore molality ($b^{\text{in-pore}}$) was calculated according to Eq. (5).

$$b^{\text{in-pore}} = \frac{moles_{CO_2}^{\text{in-pore}} + moles_{HCO_3^-}^{\text{in-pore}}}{m_{\text{water}}^{\text{in-pore}}} \tag{5}$$

The mass of in-pore water ($m_{\text{water}}^{\text{in-pore}}$) was calculated from m_{water} using Eq. (6), based on the integrals of the in-pore and ex-pore water peaks ($I^{\text{in-pore}}$ and $I^{\text{ex-pore}}$ respectively) in the ^{1}H NMR spectrum.

$$m_{\text{water}}^{\text{in-pore}} = m_{\text{water}} \times \frac{I^{\text{in-pore}}}{I^{\text{in-pore}} + I^{\text{ex-pore}}} \tag{6}$$

Calculation of solubility enhancements

Solubility enhancements (SE) for all cases were calculated according to Eq. (7) by dividing the calculated molality for the activated carbon/solvent system (b) by the literature figure for CO_2 molality in that solvent under standard conditions (b_{bulk}).[27,28] At 1 atm of pressure, this was 19.6 μmol g^{-1} for 1 M $Na_2SO_{4(aq)}$ and 32.9 μmol g^{-1} for deionized water.

$$SE = \frac{b}{b_{bulk}} \tag{7}$$

Species-specific solubility enhancements (SE_{CO_2} and $SE_{HCO_3^-}$) and in-pore solubility enhancements ($SE^{\text{in-pore}}$) were calculated from the equivalent species-specific molality (b_{CO_2} and $b_{HCO_3^-}$) or in-pore molality ($b^{\text{in-pore}}$).

Adjusting b_{bulk} for Henry's Law

b is measured when p_{CO_2} is 0.7 atm of pressure. The literature values used for CO_2 solubility[27,28] are meanwhile reported for p_{CO_2} equal to 1 atm of pressure. The b_{bulk} values used in Eq. (7) have therefore been reduced proportionally to 70% of the values given in the literature based on Henry's Law (Eq. (8)), which states that at equilibrium the molality of a dissolved species in the aqueous phase (b) is proportional to the partial pressure of the species in the gas phase (p).

$$H_s^{bp} = \frac{b}{p} \tag{8}$$

Without applying this correction, solubility enhancements remains high (see Table S2), indicating that even if b behaves non-linearly with regards to p_{CO_2}, the oversolubility effect is still significant.

Gas sorption experiments

CO_2 sorption isotherms were collected on an Autosorb iQ volumetric gas adsorption analyzer at 25 °C using a circulating water bath. Samples were activated at 100 °C in vacuum for 15 hrs before gas sorption measurements.

Temperature-programmed desorption

Temperature-programmed desorption (TPD) measurements were performed in a home-made setup consisting of a reaction unit and a gas analysis unit, as described in previous work.[29] The reaction unit consists of a high-frequency induction heating system (EASYHEAT 8310LI, Ambrell), a graphite holder inside a quartz chamber with circulating cooling water and a radiation thermometer to measure the holder temperature. A holder made of high-purity graphite (PYROGRAPH, Toyo Tanso, 99.9999%) was used. In this work, the temperature of the holder was measured from the bottom side through a quartz window using the new setup reported in our recent work to improve the sensitivity of temperature detection.[30] The gas analysis unit consists of a quadrupole mass spectrometer (QMS, MPH-100M, Inficon Co., Ltd.), a gas reservoir for calibration, and a stainless steel high-vacuum line pumped by turbo molecular pump (TMP).

Before each TPD test, the empty sample holder was heat treated at 1800 °C under high vacuum for 1 hour to clean the surface and ensure that no quantifiable amounts of gas were released during the subsequent TPD test. The weight of the activated carbon sample (about 1-2 mg) for each TPD test was measured using a high-precision electronic balance (Sartorius SE2) and placed in the graphite sample holder. Then the reaction unit was vacuumed to 2×10^{-6} Pa and heated from ambient temperature to 1800 °C at 10 °C per minute. The gases (H_2, H_2O, CO and CO_2) released during heating were quantified using a calibrated QMS where calibration gases are supplied from a gas reservoir to establish a response curve correlating the intensity of the detector signal with known gas concentrations.

TPD data has also been used to quantify the number of edge sites in different carbon samples, in particular the edge terminations by hydrogen and oxygen.[31] In addition, the average TPD domain size of graphene in nanometers (G) can be estimated using Eq. (9),[32] assuming that the shape of the graphene sheets is corona-type hexagonal and the edge sites are on the very outside.[30]

$$G = \frac{a_0}{6N_{edge}} \tag{9}$$

a_0 corresponds to the lattice parameter of the a-axis (0.2461 nm) and N_{edge} corresponds to the total number of edge sites per carbon weight (in moles per gram). N_{edge} is calculated based on the amounts of desorbed species ($N_{species}$) according to Eq. (10).

$$N_{edge} = 2N_{H_2} + 2N_{H_2O}^{>400^oC} + N_{CO} + N_{CO_2} \tag{10}$$

pH-drift measurements of ACC-10 and ACC-20/1 M $Na_2SO_{4(aq)}$ suspensions

Measurements were carried out by adding 12.5 mg of washed activated carbon into 1 mL of a series of 1 M $Na_2SO_{4(aq)}$ stock electrolytes prepared at a different pH value ranging from 1.5 to 12.5. The pH value of each stock electrolyte was adjusted by adding small quantities of either HCl_{aq} or $NaOH_{aq}$ solution as appropriate. Samples were sealed and left to equilibrate for 24 hours before measuring the pH (*pH (final)* in Fig. S13) with a pH meter (905 Titrando, Metrohm). An additional set of electrolytes at the same pH values without added carbon was prepared to control for uptake of atmospheric $CO_{2(g)}$. pH was then measured and used as *pH (initial)* in Fig. S13.

Computational details

Computational simulations were enabled through the use of MACE potentials.[33] These were trained on a representative selection of structures labeled with energies and forces calculated at the DFT level. Structures were generated using a variety of methods, including *ab initio* MD, MACE-MD, enhanced sampling simulations, and constrained MD runs. The resulting dataset, optimized using the procedure outlined in Schran et al.,[34] totaled some 9000 structures. A variety of systems were included: pure water, CO_2 in water, nanoconfined systems,

and reactive CO_2 trajectories. Structures were labeled with DFT energies and forces calculated using the QUICKSTEP method of `CP2K`.[35,36] The revPBE functional augmented by Grimme's D3 corrections[37–39] was chosen on account of its ability to reproduce the structural properties of bulk and interfacial water.[40–43] Goedecker–Teter–Hutter (GTH) pseudopotentials were selected for the treatment of core electrons, whilst the TZV2P-GTH (O and H) and DZVP-MOLOPT-SR-GTH (C) basis sets were chosen for valence electron density. We selected an auxiliary plane-wave cutoff of 1200 Ry.

Using this optimized dataset, a two-layer MACE model with 128 equivariant messages was generated. We selected a maximal message equivariance of L = 1 and a radial cutoff of $R_\mathrm{c} = 5$ Å, equating to an effective receptive field of 10 Å after message passing. Validation of the model was performed to assess the accuracy of its force, energy, and structural predictions (Figs. S15 to S16). We report RMSEs of 1.2 meV/atom for the energies and 41.8 meV/Å for the forces. Radial distribution functions (RDFs) were generated for pure water and CO_2-water systems and compared with *ab initio*-MD and experimental RDFs. The results of this analysis, shown in Fig. S16, demonstrate the accuracy of our model for reproducing the properties of bulk aqueous systems.

Free MD simulations

Density profiles for H_2O, CO_2, and HCO_3^- were generated using unconstrained molecular dynamics simulations. We approximated the pore environment by using a nanoconfined aqueous system, with the flexible graphene sheets separated by 7 Å, 12 Å, and 15 Å. Details of the system setups are given in Table S1 below.

Molecular dynamics simulations were run using `LAMMPS`.[44,45] Simulations were performed using the NVT ensemble at 300 K. The Nosé-Hoover thermostat was selected with a time constant of 100 fs. Deuterium masses were used for the hydrogen atoms along with a timestep of 1 fs. Convergence of the density profiles was monitored using block averaging, and each trajectory accumulated over several ns of simulation time. The resulting profiles are shown

in Fig. 4a of the main text. An additional profile obtained for 20 Å pore separation (see Table S1, System 4) is shown in Fig. S17.

In Fig. 4d of the main text, we detail orientational analyses of the molecules residing at the pore-water contact layer. For these analyses, we utilized structures extracted from the 12 Å pore trajectory (used in Fig. 4a). Orientations were measured by the angle between the z axis and either the H_2O dipole vector or CO_2's bond vector. Only the orientations of species adjacent to one pore wall were considered. Snapshots of this contact layer (omitting species beyond the first layer) are also shown in Fig. 4e.

Table S1: System setups for the idealized pore environments used in this work. Systems highlighted in bold were used for obtaining the density profiles shown in Fig. 4a. An additional free MD simulation was performed using System 4, the results of which can be found in Fig. S17. System 5 was used for obtaining the aqueous free energy profiles shown in Fig. 4c of the main text.

	Pore Width	Cell Dimensions (Å)	n(CO_2/HCO_3^-)	n(H_2O)
**System 1**	7.0	12.35×12.834×31.0	1	14
**System 2**	12.0	17.29×17.112×35.0	1	81
**System 3**	15.0	17.29×17.112×40.0	1	115
System 4	20.0	17.29×17.112×35.0	1	157
System 5	30.0	12.35×12.834×55.0	1	140

Gaseous interaction energies

In Fig. 4b, we report the interaction energies for gaseous CO_2 and H_2O interacting with an isolated graphene sheet (5×5 supercell, 20 Å vacuum). Estimates of the interaction energy, E_{int}, were obtained from single-point DFT calculations of CO_2/H_2O located at different distances from the graphene plane. To calculate E_{int}, we used the following formula,

$$E_{\text{int}} = E[\text{Molec.} + \text{wall}] - E[\text{Molec.} - \text{wall}], \tag{11}$$

where E[Molec. + wall] gives the energy of the configuration in which CO_2/H_2O is adsorbed at the pore wall, and E[Molec. − wall] is the energy of the configuration where CO_2/H_2O is located 10 Å away (rigid translation). revPBE-D3 calculations were performed with VASP[46–49] using the projector-augmented plane wave method and hard pseudo-potentials[50,51] (energy cutoff of 1000 eV). A 1×1×1 k-point grid was used to sample the Brillouin zone, providing converged energies to within 1 meV of a 4×4×1 grid.

Aqueous free energies of adsorption

In Fig. 4c, we report the free energies of both CO_2 and H_2O as a function of the distance from the pore wall under pore-saturated conditions. To generate these profiles, we utilized a 30 Å pore environment (System 5), the details of which can be found in Table S1. The H_2O free energy curve was generated from a Boltzmann inversion of the underlying water density profile,

$$\Delta F = -RT \ln(\rho/\rho_0), \tag{12}$$

where T is the temperature, R is the gas constant, ρ is the density, and ρ_0 is the bulk density (CO_2 was removed from these runs to prevent artifacts in the profile). For CO_2, given the difficulty associated with obtaining converged statistics for a single molecule, free energies were obtained using the umbrella integration method.[52] The $\sim$ 15 Å range of pore-CO_2 distances was divided into 50 separate windows. For each window, a harmonic restraint with force constant k = 10 kcal mol^{-1} Å^{-2} was applied. Each window was simulated for 400 ps. Errors of integrations are shown alongside free energies in the main plot. The H_2O and CO_2 free energies are aligned such that the energy zero is set at 15 Å (i.e., bulk) for both molecules.

Supplementary Tables

Table S2: Calculated molalities (b) and solubility enhancements (SE) for CO_2-dosed activated carbon/aqueous solvent systems. b is the molality of dissolved CO_2-derived species in mol kg^{-1}, with b_{CO_2} referring to only CO_2. $b^{\text{in-pore}}$ is the molality when considering only the in-pore mass of the aqueous solvent (based on integration of in-pore and ex-pore signals in the ^{1}H NMR spectrum). Both are given in units of mmolg^{-1}. SE and $SE^{\text{in-pore}}$ are calculated from b and $b^{\text{in-pore}}$ respectively as described in Eq. (7). SE^{HL} is calculated in the same manner at SE, but without accounting for Henry's Law (see Eq. (8) and discussion).

AC	ACC-10	ACC-20	ACC-20	AEL-1200	ACC-10	ACC-20
Solution	1 M $Na_2SO_{4(aq)}$				DI water	
v:m (mg:µL)	1:1	1:1	2:3	2:3	1:1	2:3
b (mmol g^{-1})	0.42	0.36	0.18	0.44	0.48	0.22
b_{CO_2} (mmol g^{-1})	0.30	0.18	0.10	0.30	0.40	0.13
SE	30.9	26.2	12.9	31.9	20.9	9.4
$b^{\text{in-pore}}$ (mmol g^{-1})	0.77	0.37	0.36	0.54	0.99	0.36
$b_{CO_2}^{\text{in-pore}}$ (mmol g^{-1})	0.54	0.18	0.21	0.37	0.83	0.22
$SE^{\text{in-pore}}$	56.1	27.0	26.3	39.6	43.0	15.5
SE^{HL}	21.6	18.4	9.1	22.3	14.6	6.6

Table S3: Literature values for BET surface area, accumulative pore volume and average pore size of carbons used in this work, calculated from N_2 sorption isotherms.[1,2]

Carbon	BET Surface Area (m^2/g)	Accumulative pore volume (cc/g)	Average pore size (Å)
ACC-10	1094	0.4	5.6
ACC-20	2004	0.8	8.9
AEL-1200	1602	0.7	9.2

Table S4: TPD results for the three studied carbons, showing elemental analysis results and average coronene model domain size (G) based on quantification of desorbed gases. Results show that ACC-10 and ACC-20 have similar compositions and are similarly disordered, while AEL-1200 contains significantly less oxygen and hydrogen and is less disordered. These measurements of carbon domain size (G) agree qualitatively with recent estimations of the domain size of activated carbons via NMR.[1]

Carbon	ACC-10	ACC-20	AEL-1200
H_2 desorbed (µmol g^{-1})	2443.1	2725.6	447.8
CO desorbed (µmol g^{-1})	619.9	380.9	129.9
CO_2 desorbed (µmol g^{-1})	110.9	60.8	27.1
H_2O desorbed (above 400 °C) (µmol g^{-1})	115.5	121.7	32.6
C at%	93.1	92.9	98.6
O at%	1.1	0.7	0.3
H at%	5.8	6.4	1.1
Total edge amount (mmol g^{-1})	5.85	6.13	1.12
SSA edge (m^2 g^{-1})	292.29	306.20	55.87
Domain size (coronene model) (nm)	7.0	6.7	36.7

Table S5: Comparison of CO_2:H_2O mole fractions observed experimentally in ACC-10 and ACC-20 + deionized water systems and CO_2 mole fractions used in atomistic modelling of graphene slit pores.

System	Type	In-pore mole fraction of CO_2 in water (%)
ACC-10/H_2O 1:1	Experimental	1.5
ACC-20/H_2O 2:3	Experimental	0.4
7 Å	Computational	6.6
12 Å	Computational	1.2
15 Å	Computational	0.9

Supplementary Figures

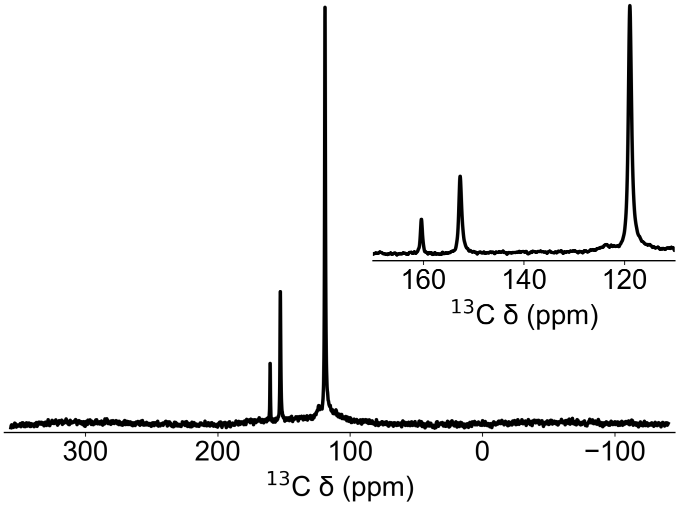

(a) ^{13}C NMR of 1:1 m/v ACC-10/1 M $Na_2SO_{4(aq)}$ dosed with $^{13}CO_{2(g)}$.

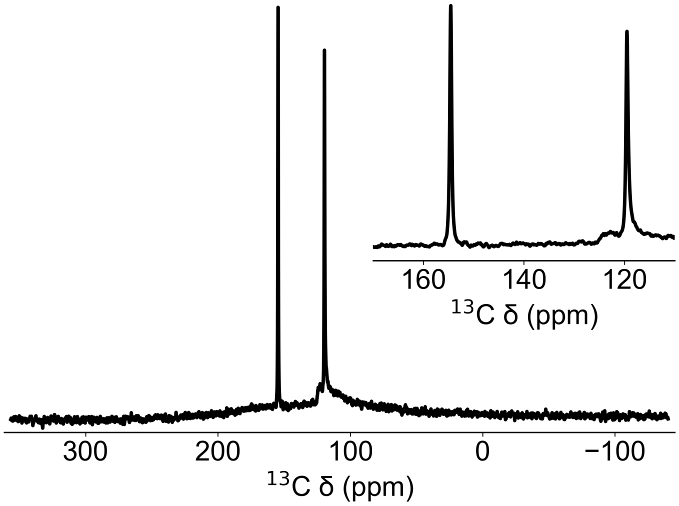

(b) ^{13}C NMR of 1:1 m/v ACC-20/1 M $Na_2SO_{4(aq)}$ dosed with $^{13}CO_{2(g)}$.

Figure S1: NMR spectra of activated carbon/solvent/$^{13}CO_{2(g)}$ systems (9.4 T, 5 kHz MAS). Insets show the same spectra across a smaller range to display peak chemical shift and shape.

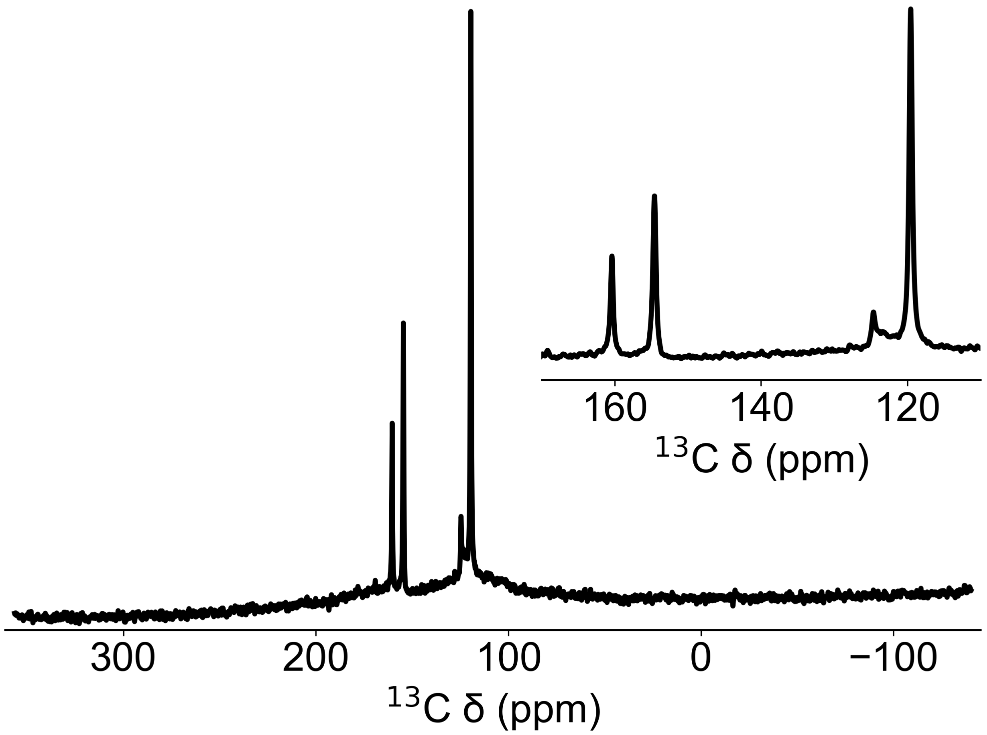

(a) ^{13}C NMR of 2:3 m/v ACC-20/1 M $Na_2SO_{4(aq)}$ dosed with $^{13}CO_{2(g)}$.

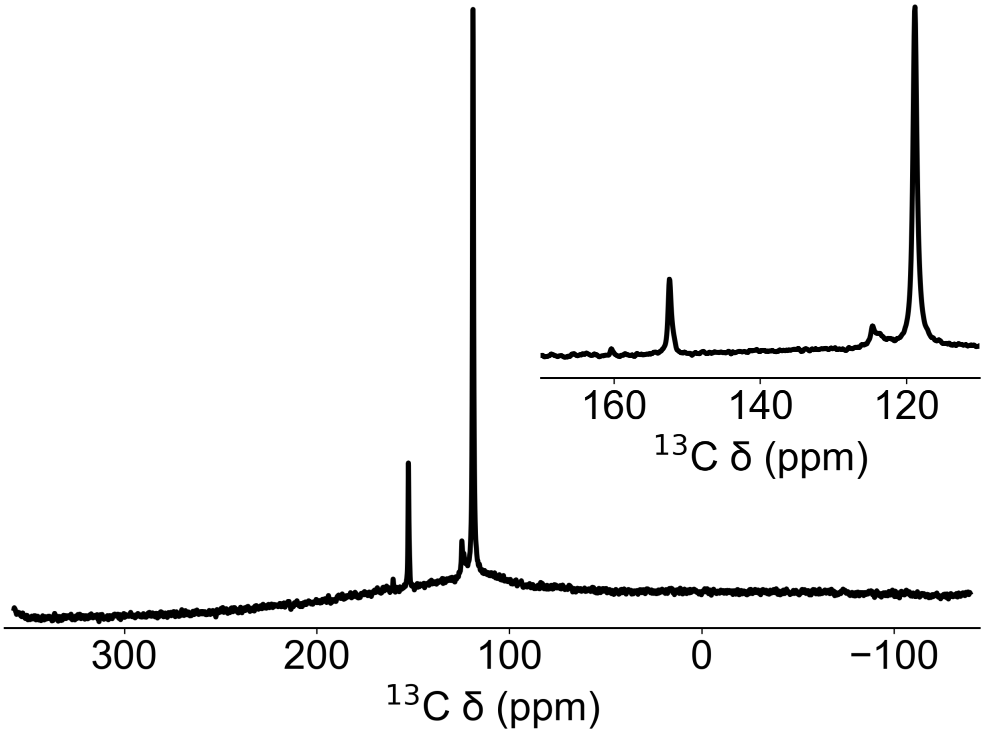

(b) ^{13}C NMR of 1:1 m/v ACC-10/DI water dosed with $^{13}CO_{2(g)}$.

Figure S2: ^{13}C NMR spectra of activated carbon/solvent/$^{13}CO_{2(g)}$ systems (9.4 T, 5 kHz MAS). Insets show the same spectra across a smaller range to display peak chemical shift and shape.

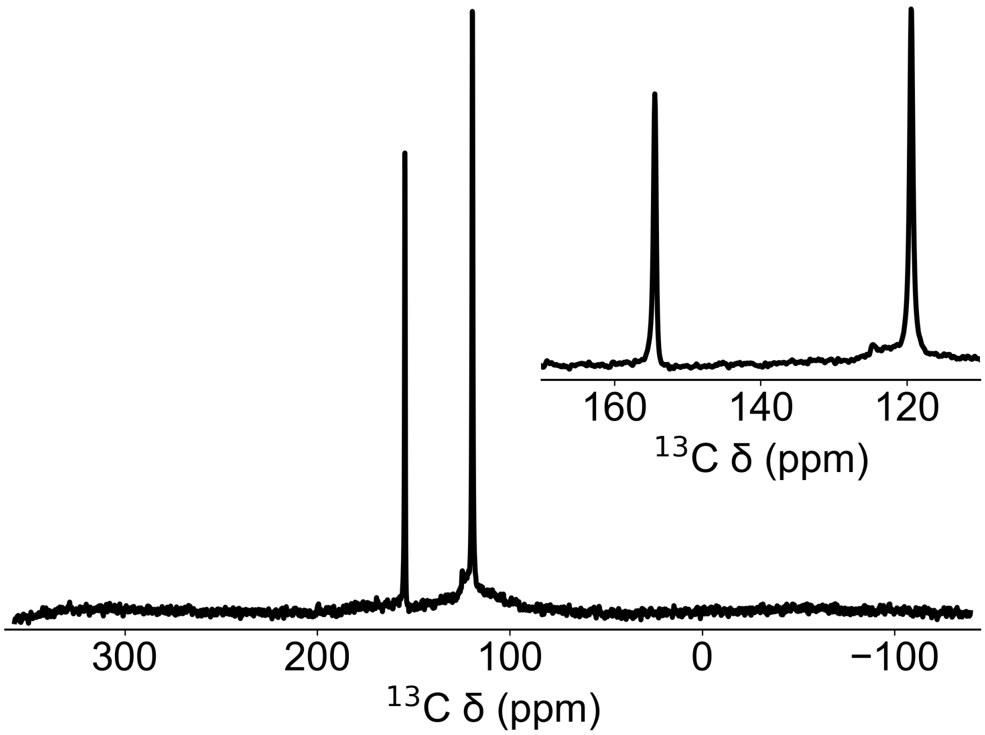

(a) ^{13}C NMR of 2:3 m/v ACC-20/DI water dosed with $^{13}CO_{2(g)}$.

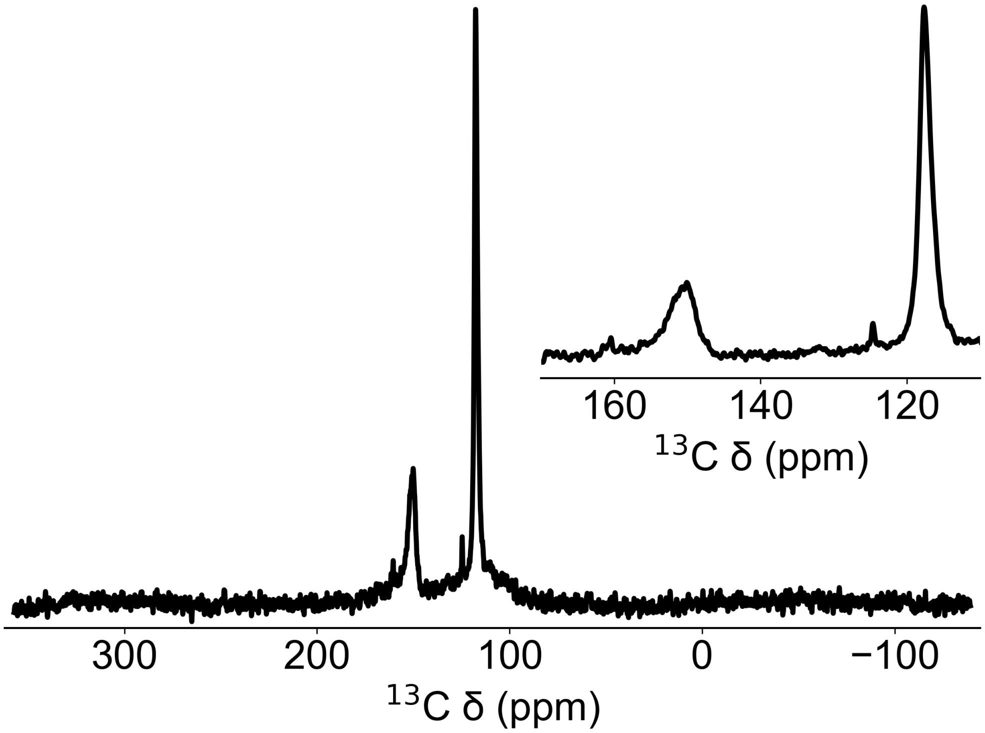

(b) ^{13}C NMR of 2:3 m/v AEL-1200/1 M $Na_2SO_{4(aq)}$ dosed with $^{13}CO_{2(g)}$.

Figure S3: ^{13}C NMR spectra of activated carbon/solvent/$^{13}CO_{2(g)}$ systems (9.4 T, 5 kHz MAS). Insets show the same spectra across a smaller range to display peak chemical shift and shape.

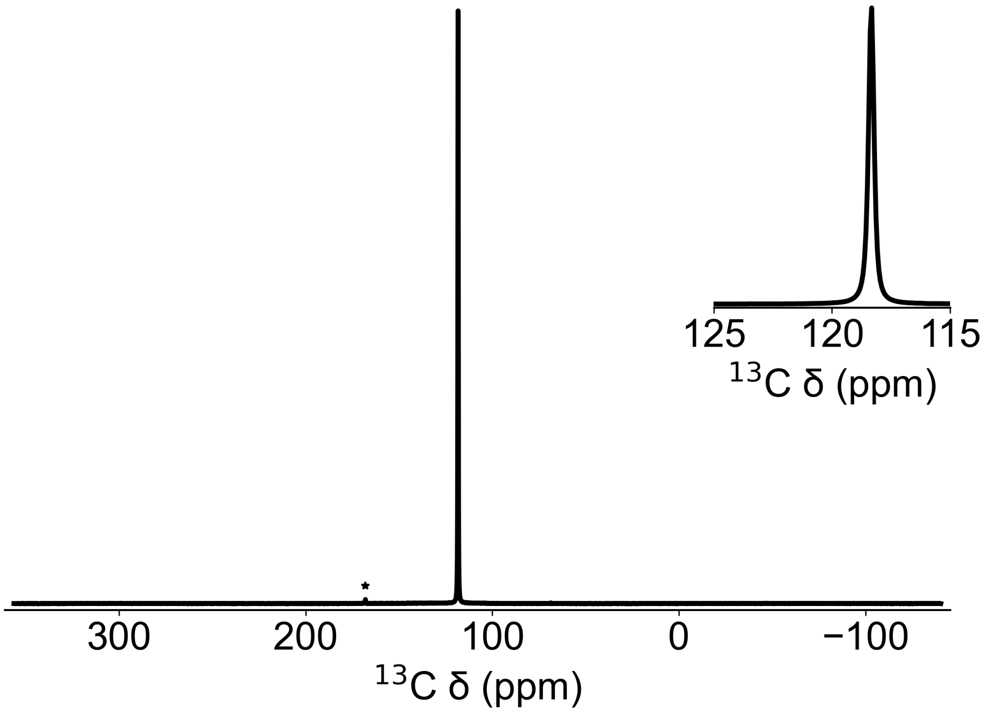

(a) ^{13}C NMR of ACC-10 dosed with $^{13}CO_{2(g)}$ at 12.9 psi of pressure. A single resonance with spinning sidebands (*) was observed.

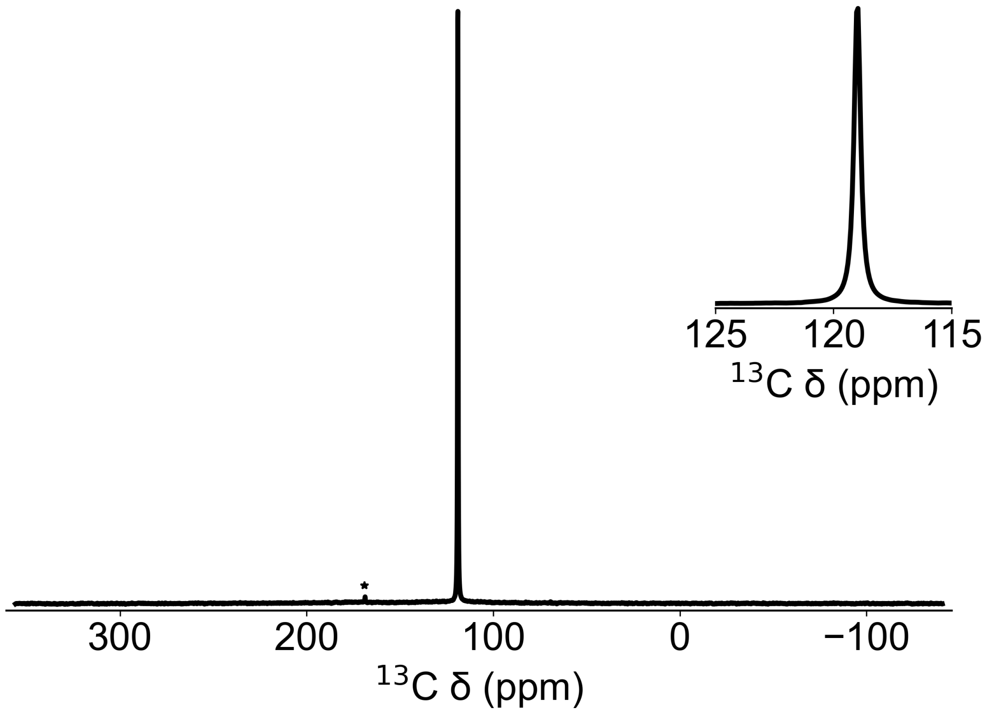

(b) ^{13}C NMR of ACC-20 dosed with $^{13}CO_{2(g)}$ at 6.4 psi of pressure. A single resonance with spinning sidebands (*) was observed.

Figure S4: ^{13}C NMR spectra of activated carbon/$^{13}CO_{2(g)}$ systems (9.4 T, 5 kHz MAS). Insets show the same spectra across a smaller range to display peak chemical shift and shape.

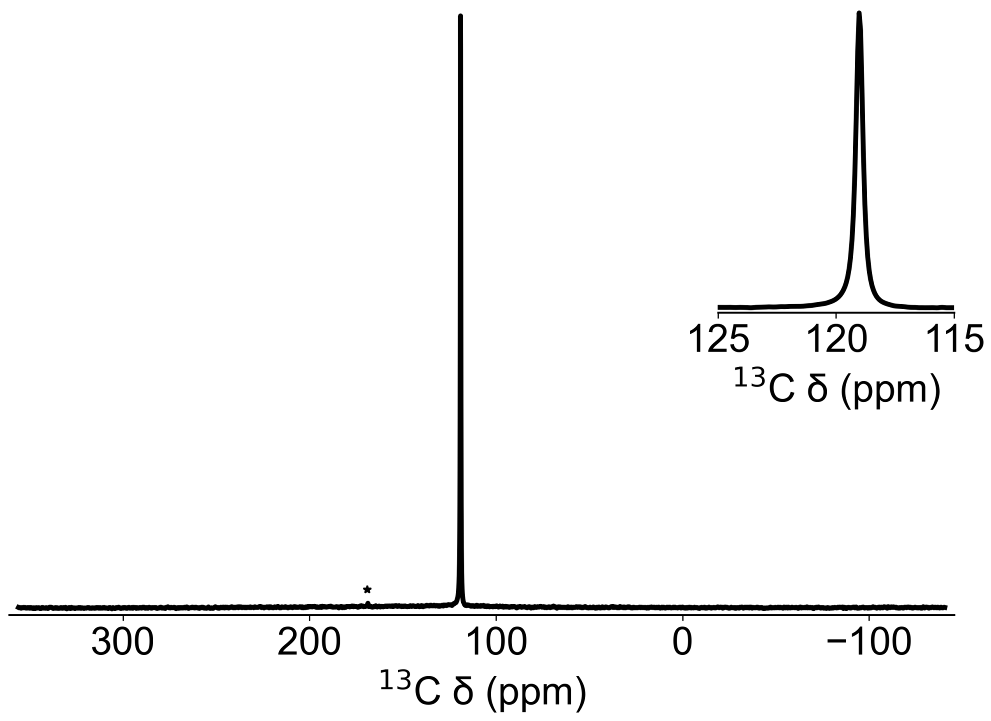

(a) ^{13}C NMR of ACC-20 dosed with $^{13}CO_{2(g)}$ at 7.6 psi of pressure. A single resonance with spinning sidebands (*) was observed.

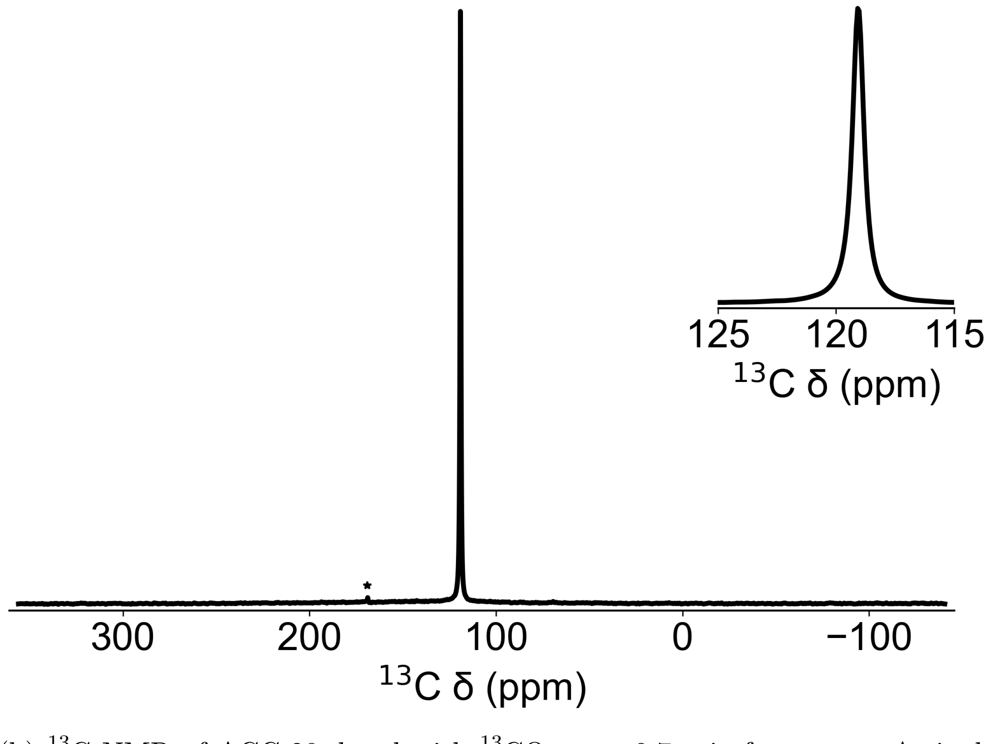

(b) ^{13}C NMR of ACC-20 dosed with $^{13}CO_{2(g)}$ at 9.7 psi of pressure. A single resonance with spinning sidebands (*) was observed.

Figure S5: ^{13}C NMR spectra of activated carbon/$^{13}CO_{2(g)}$ systems (9.4 T, 5 kHz MAS). Insets show the same spectra across a smaller range to display peak chemical shift and shape.

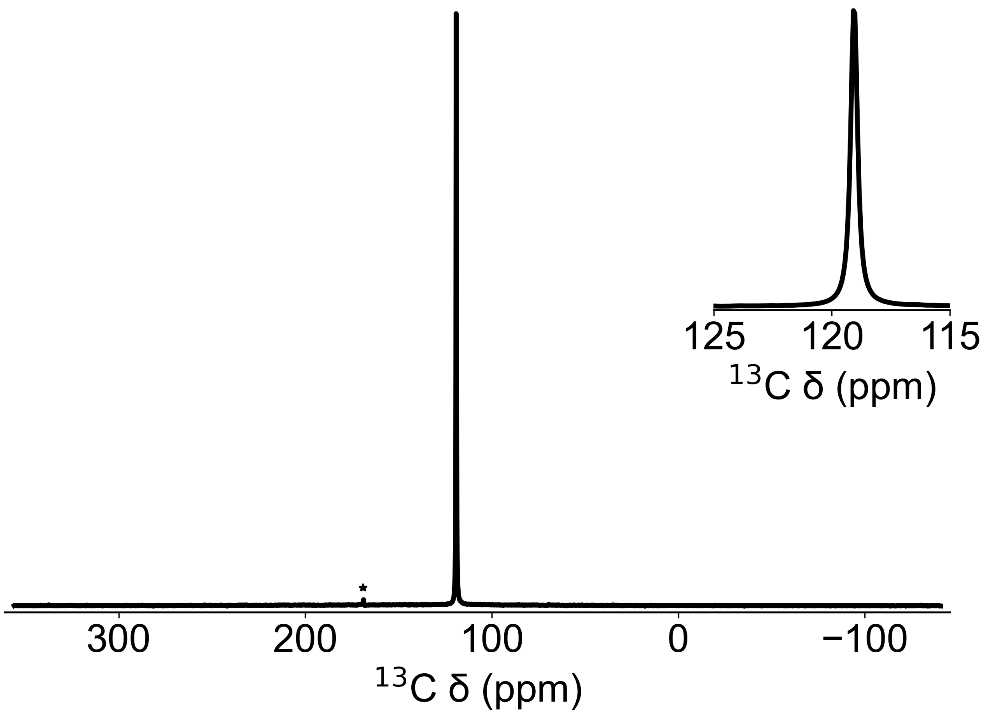

(a) ^{13}C NMR of ACC-20 dosed with $^{13}CO_{2(g)}$ at 11.3 psi of pressure. A single resonance with spinning sidebands (*) was observed.

Figure S6: ^{13}C NMR spectra of activated carbon/$^{13}CO_{2(g)}$ systems (9.4 T, 5 kHz MAS). Insets show the same spectra across a smaller range to display peak chemical shift and shape.

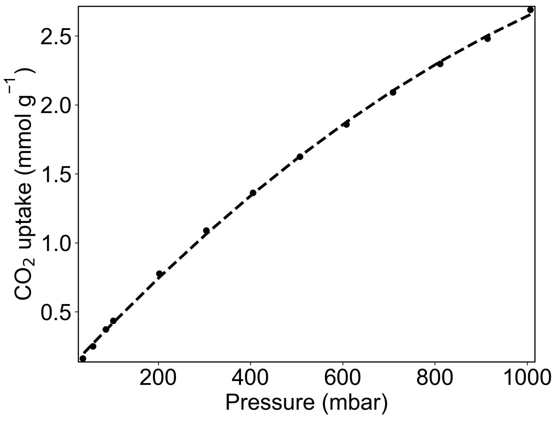

(a)

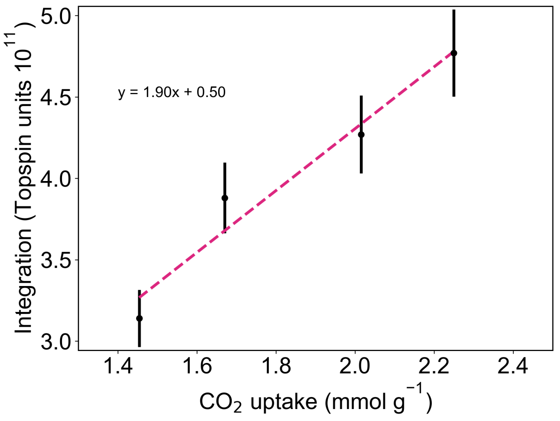

(b)

Figure S7: a) Volumetric gas sorption isotherm for CO_2 uptake in ACC-20, measured at 25 °C using a circulating water bath. Samples were activated at 100 °C in vacuum for 15 hrs before gas sorption measurements. Points were fitted to the Langmuir adsorption equation to estimate CO_2 uptake at pressures between measured data points.[53] b) Calibration curve for integration signal of the adsorbed CO_2 peak in $^{13}CO_{2(g)}$-dosed ACC-20 samples at variable pressure, plotted against CO_2 uptake expected at that pressure from the gas sorption isotherm. Error bars reflect the 5.6% standard deviation/mean value previously calculated for multiple measurements of the in-pore $^{13}CO_2$ peak. Inclusion of a constant in the linear fit is expected due to additional ^{13}C signal from the activated carbon.

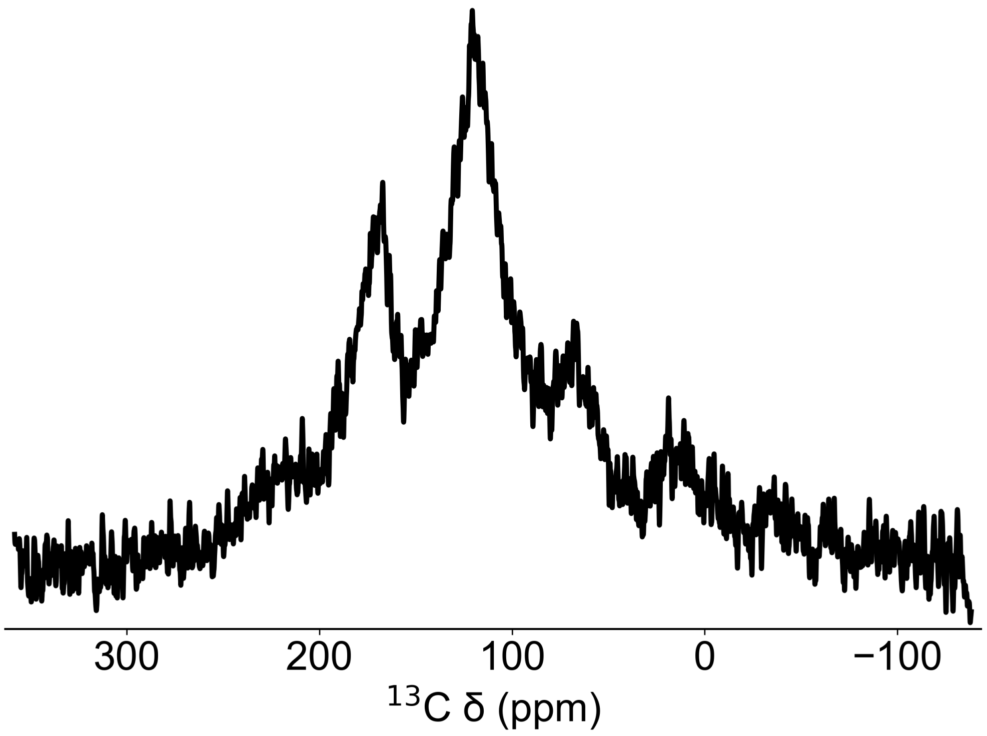

(a) ^{13}C NMR of ACC-10.

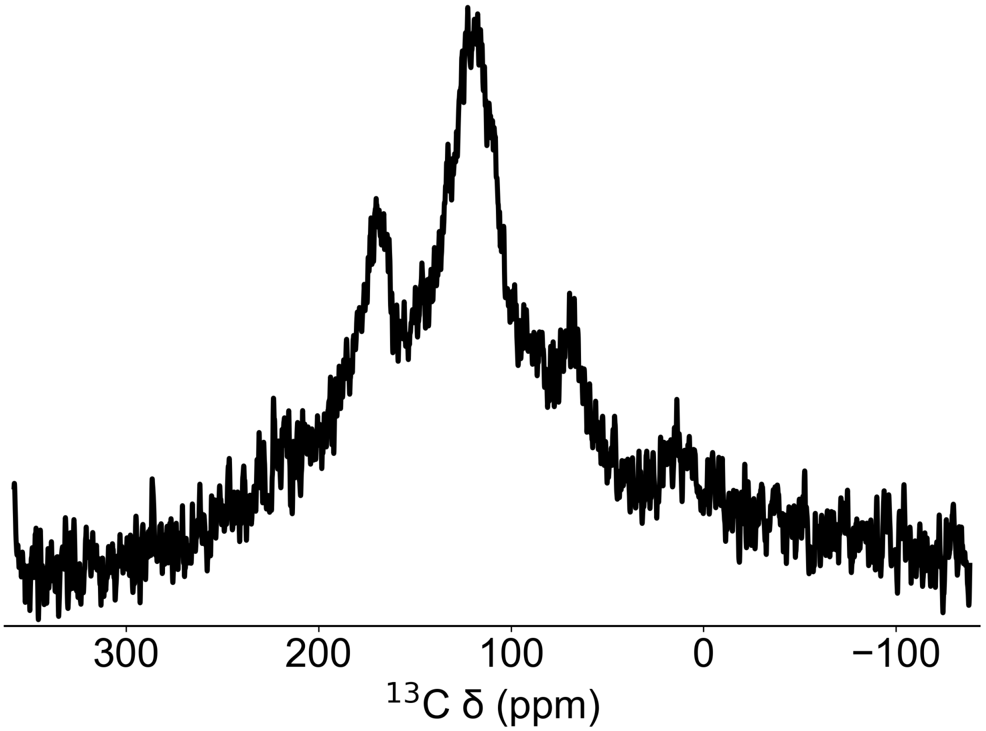

(b) ^{13}C NMR of ACC-20.

Figure S8: ^{13}C NMR spectra of activated carbon and activated carbon/solvent systems (9.4 T, 5 kHz MAS).

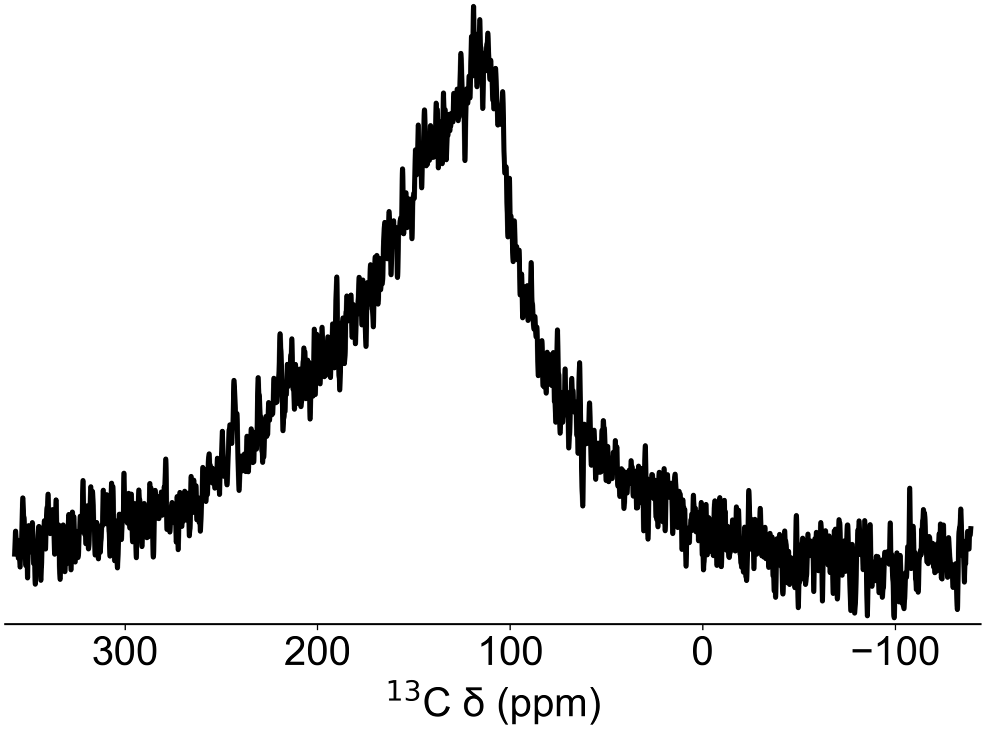

(a) ^{13}C NMR of AEL-1200.

Figure S9: ^{13}C NMR spectra of activated carbon and activated carbon/solvent systems (9.4 T, 5 kHz MAS).

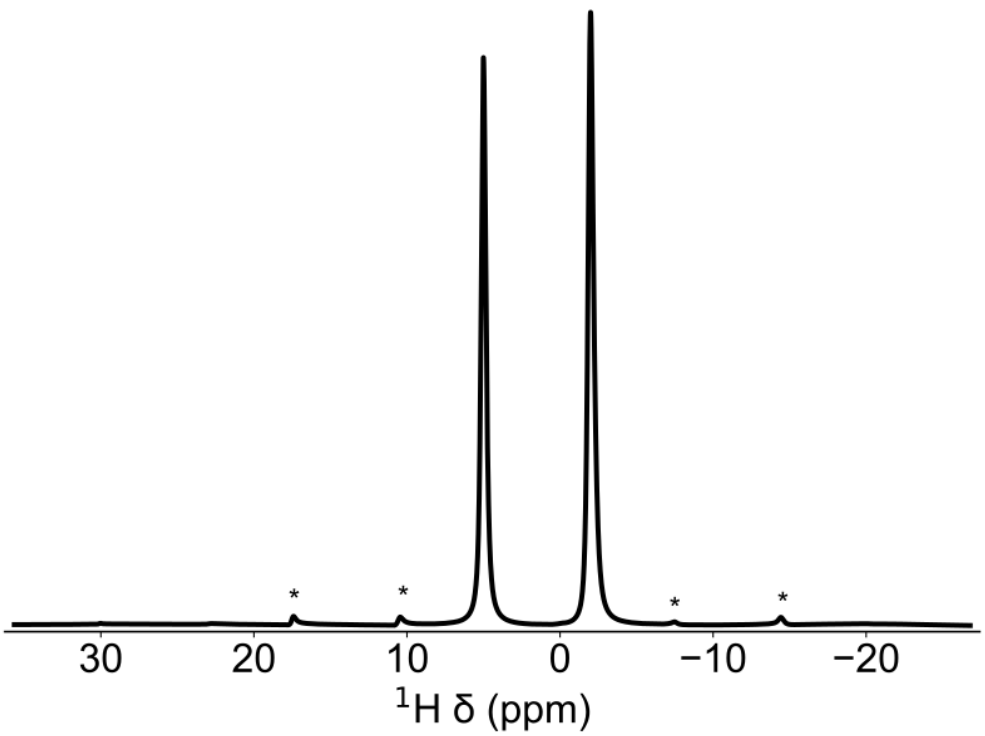

(a) ^{1}H NMR of 1:1 m/v ACC-10/1 M $Na_2SO_{4(aq)}$ dosed with $^{13}CO_{2(g)}$. Spinning sidebands are marked with *.

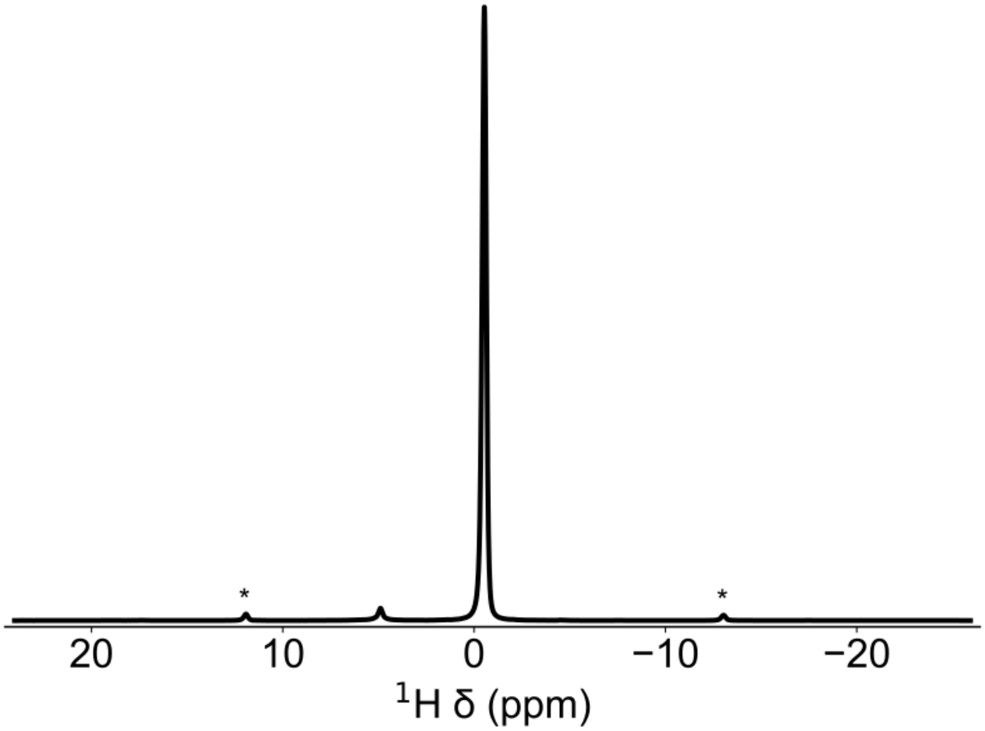

(b) ^{1}H NMR of 1:1 m/v ACC-20/1 M $Na_2SO_{4(aq)}$ dosed with $^{13}CO_{2(g)}$. Spinning sidebands are marked with *.

Figure S10: ^{1}H NMR spectra of activated carbon/solvent/$^{13}CO_{2(g)}$ systems (9.4 T, 5 kHz MAS).

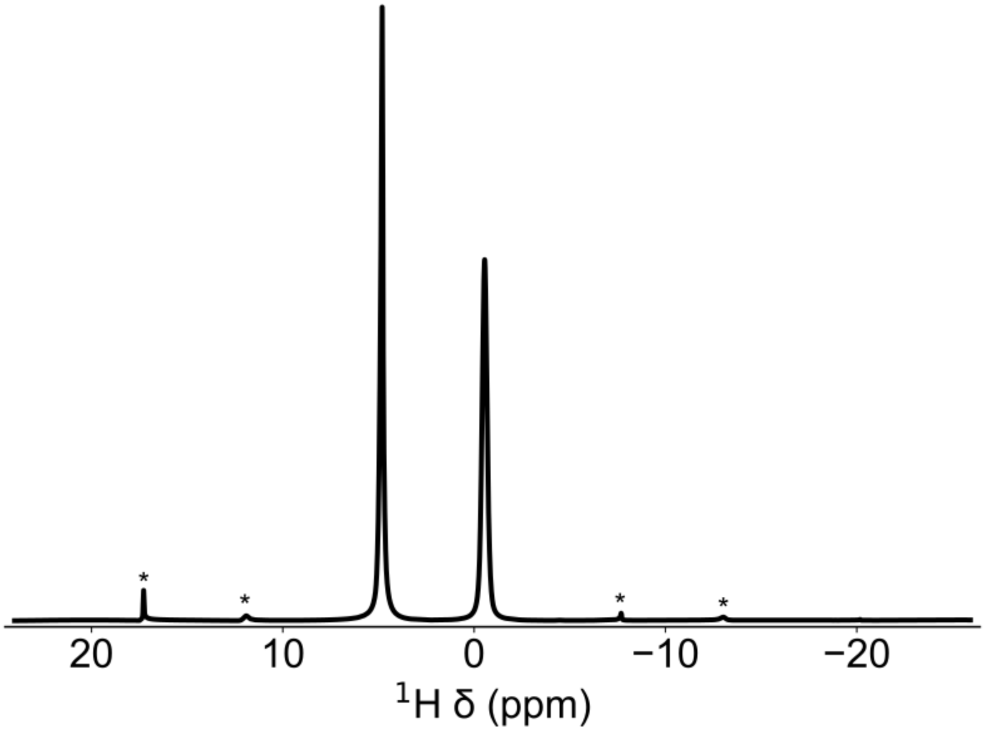

(a) ^{1}H NMR of 2:3 m/v ACC-20/1 M $Na_2SO_{4(aq)}$ dosed with $^{13}CO_{2(g)}$. Spinning sidebands are marked with *.

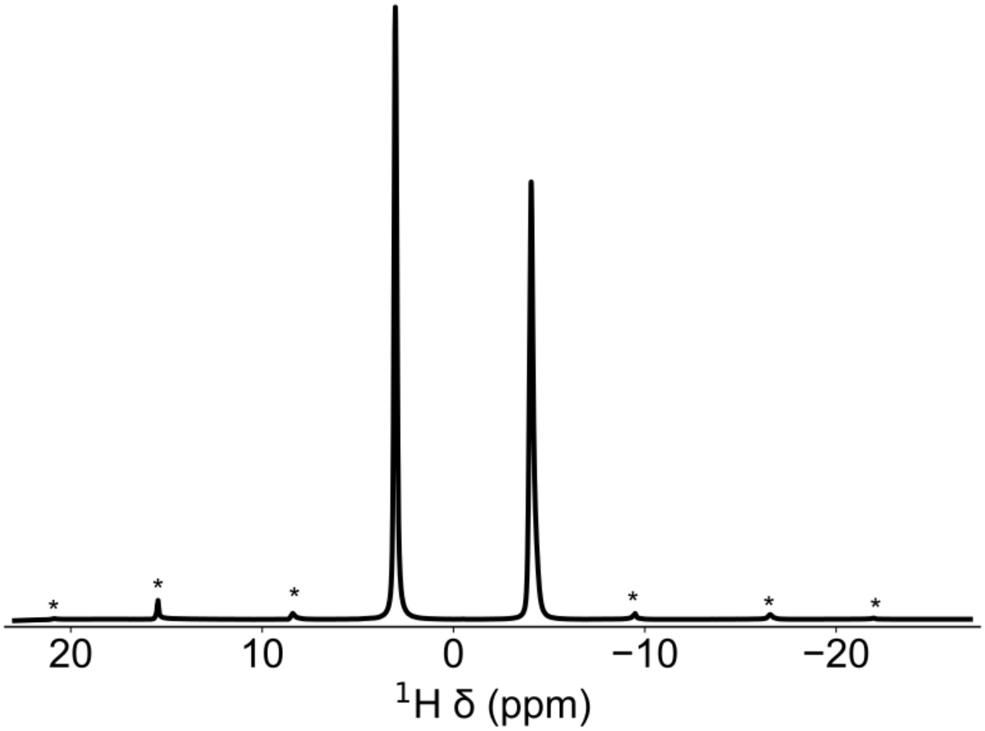

(b) ^{1}H NMR of 1:1 m/v ACC-10/DI water dosed with $^{13}CO_{2(g)}$. Spinning sidebands are marked with *.

Figure S11: ^{1}H NMR spectra of activated carbon/solvent/$^{13}CO_{2(g)}$ systems (9.4 T, 5 kHz MAS).

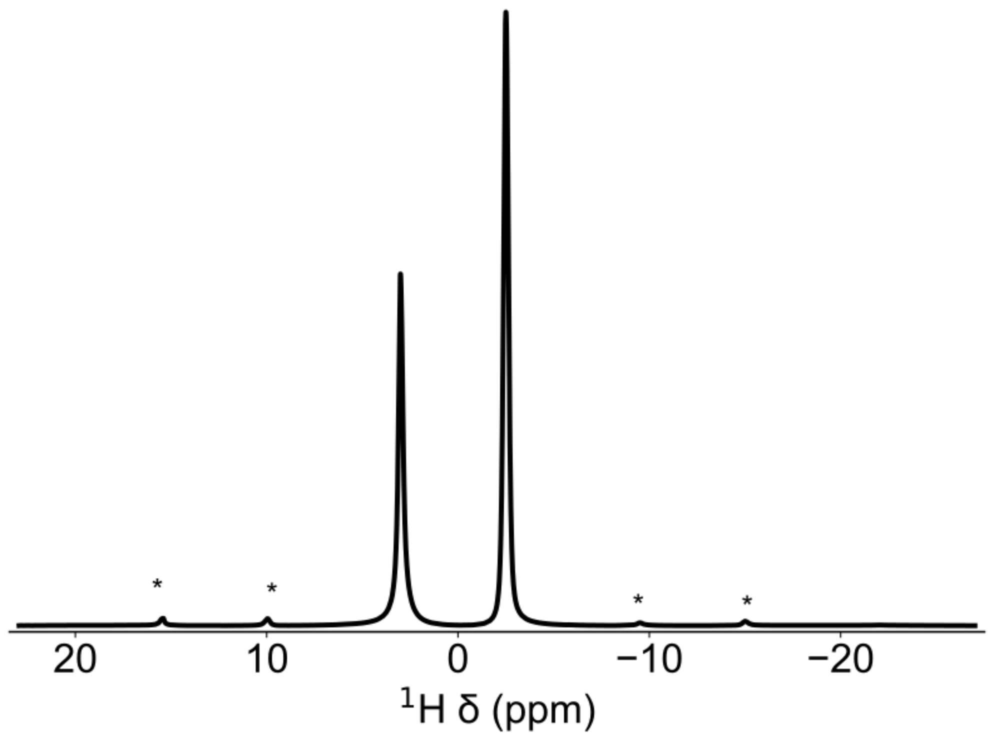

(a) ^{1}H NMR of 2:3 m/v ACC-20/DI water dosed with $^{13}CO_{2(g)}$. Spinning sidebands are marked with *.

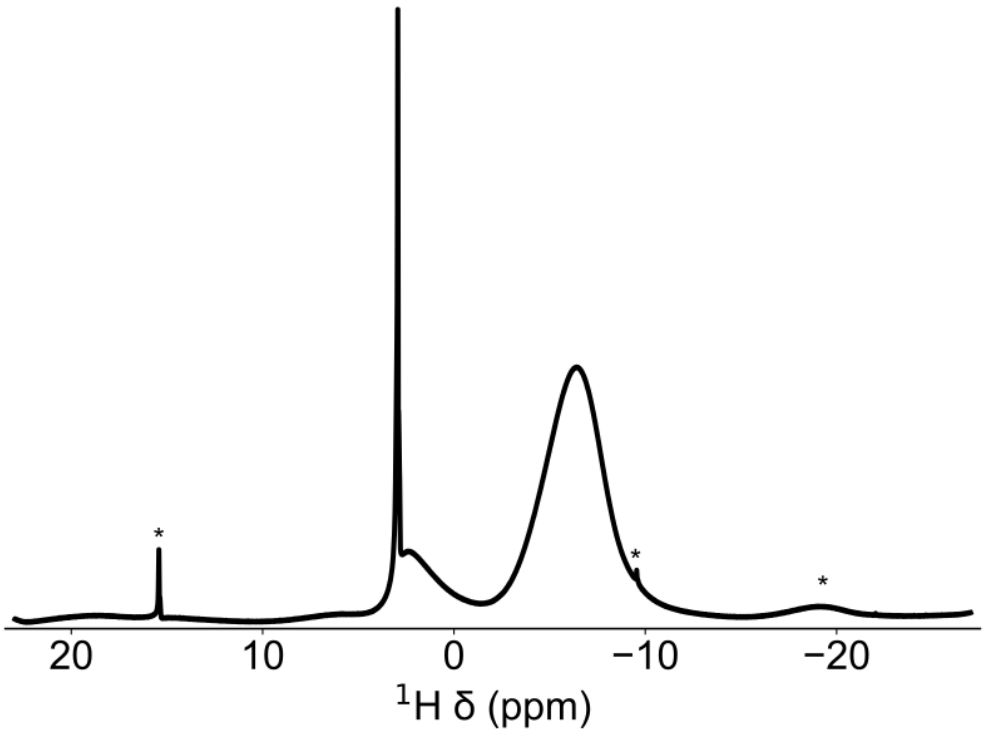

(b) ^{1}H NMR of 2:3 m/v AEL-1200/1 M $Na_2SO_{4(aq)}$ dosed with $^{13}CO_{2(g)}$. Spinning sidebands are marked with *.

Figure S12: ^{1}H NMR spectra of activated carbon/solvent/$^{13}CO_{2(g)}$ systems (9.4 T, 5 kHz MAS).

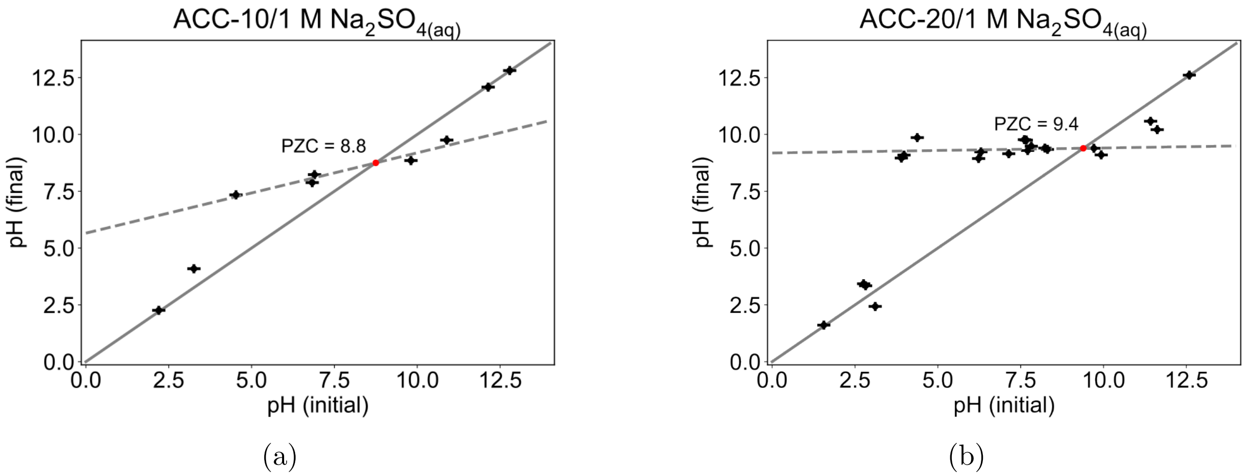

Figure S13: pH-drift measurements of the activated carbon/1 M $Na_2SO_{4(aq)}$ system demonstrate that both carbons have a basic surface. *pH (initial)* reflects the pH of the liquid phase no in contact with the activated carbon, while *pH (final)* reflects the pH of the liquid phase in contact with the activated carbon. Dashed lines draw attention to the plateau region over which the activated carbon affects the solution pH significantly.

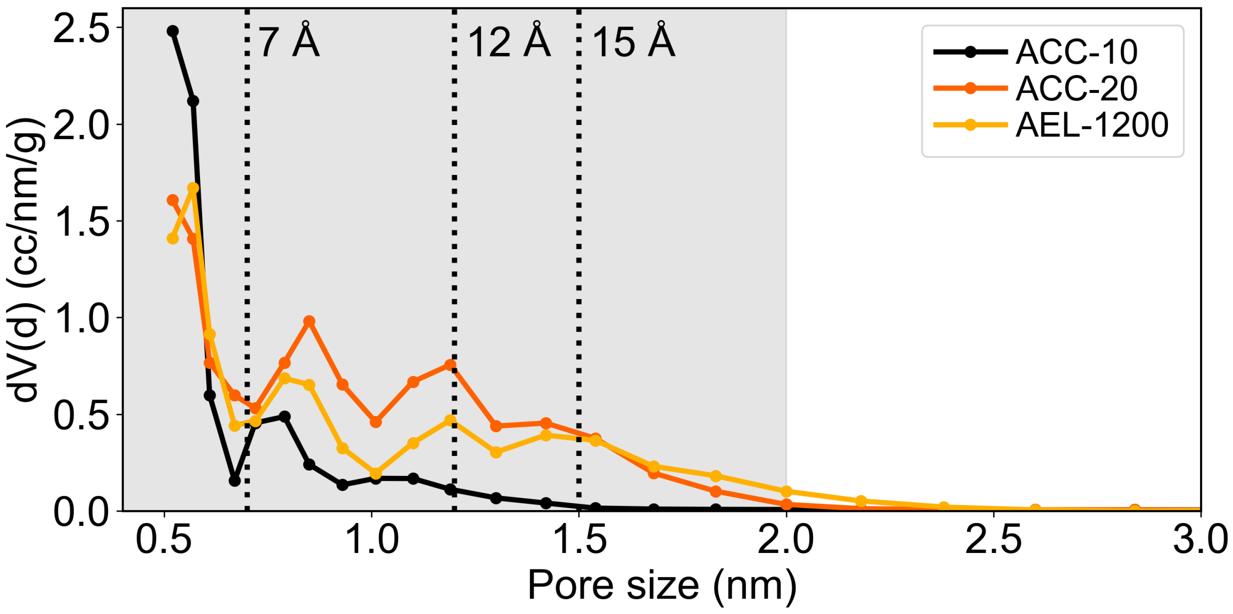

Figure S14: Previously reported pore size distributions of ACC-10, ACC-20, and AEL-1200 as measured by N_2 sorption isotherm analysis.[1,2] Dotted lines correspond to the sizes of pores studied in Fig. 4a. The grey area represents the microporous region.

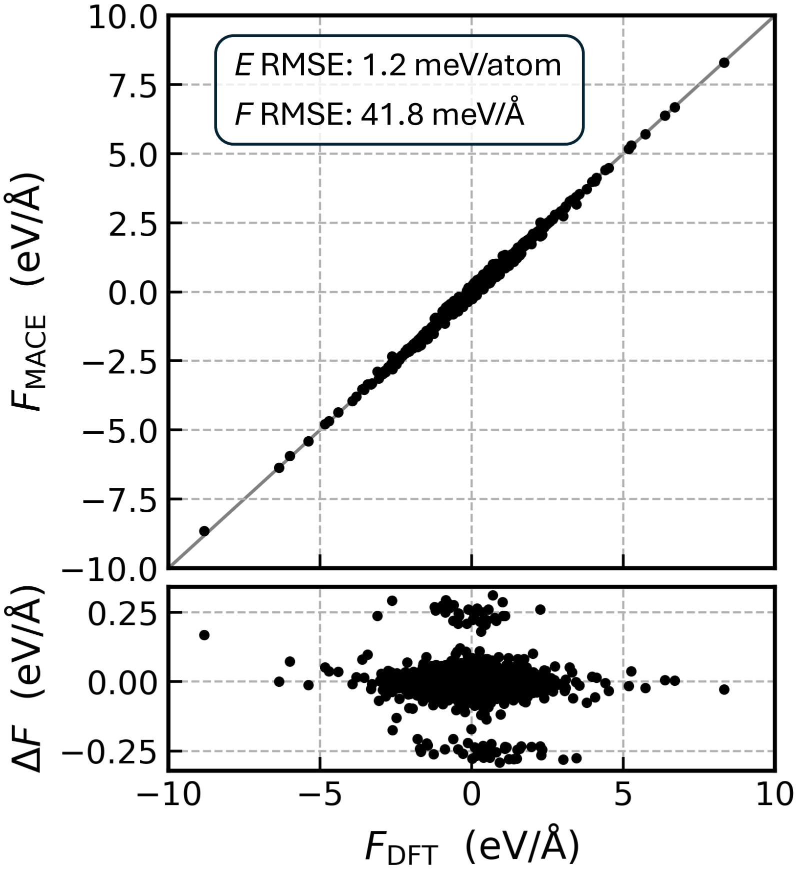

Figure S15: A comparison of MACE and DFT forces calculated for a subset of structures extracted from the main training dataset. (**Top**) Forces calculated using our MACE potential plotted against those of revPBE-D3. (**Bottom**) The difference in MACE and revPBE-D3 forces (ΔF) plotted against revPBE-D3 forces. Overall model RMSEs for the energies and forces are shown at the top of the plot.

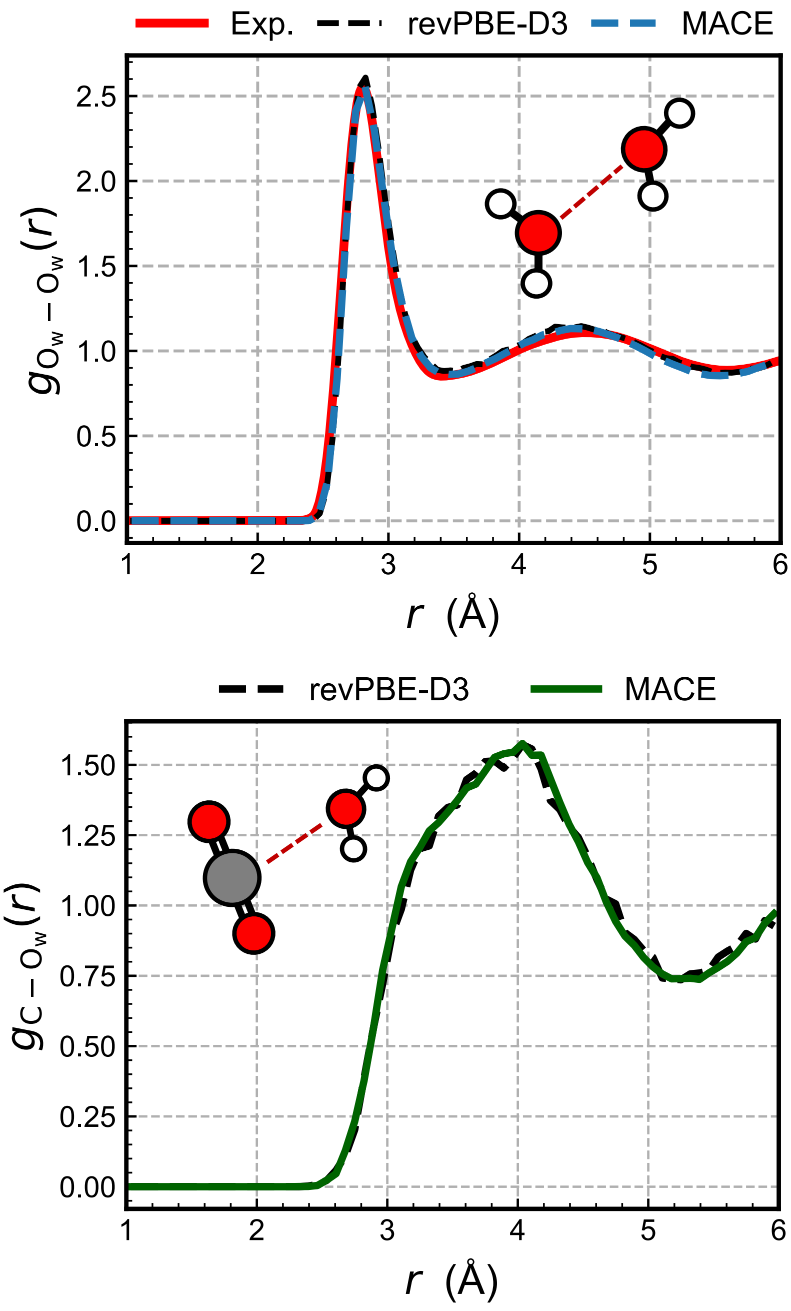

Figure S16: Radial distribution function (RDF) predictions of our MACE model compared with those of DFT (revPBE-D3). (**Top**) Ow-Ow RDFs obtained from MACE, revPBE-D3, and experiment. MACE RDFs were obtained using a 64-molecule water system with equal side lengths of 12.42 Å. MACE simulations were performed over 1 ns and the results compared to those of *ab initio*-MD obtained using identical system setups. Experimental results were taken from x-ray diffraction data.[54,55] (**Bottom**) C-Ow RDFs compared for MACE and revPBE-D3. RDFs were obtained using a 63-molecule water system with a single CO_2 molecule and equal side lengths of 12.57 Å. MACE simulations were performed over 1 ns and the results compared to those of *ab initio*-MD obtained using identical system setups.

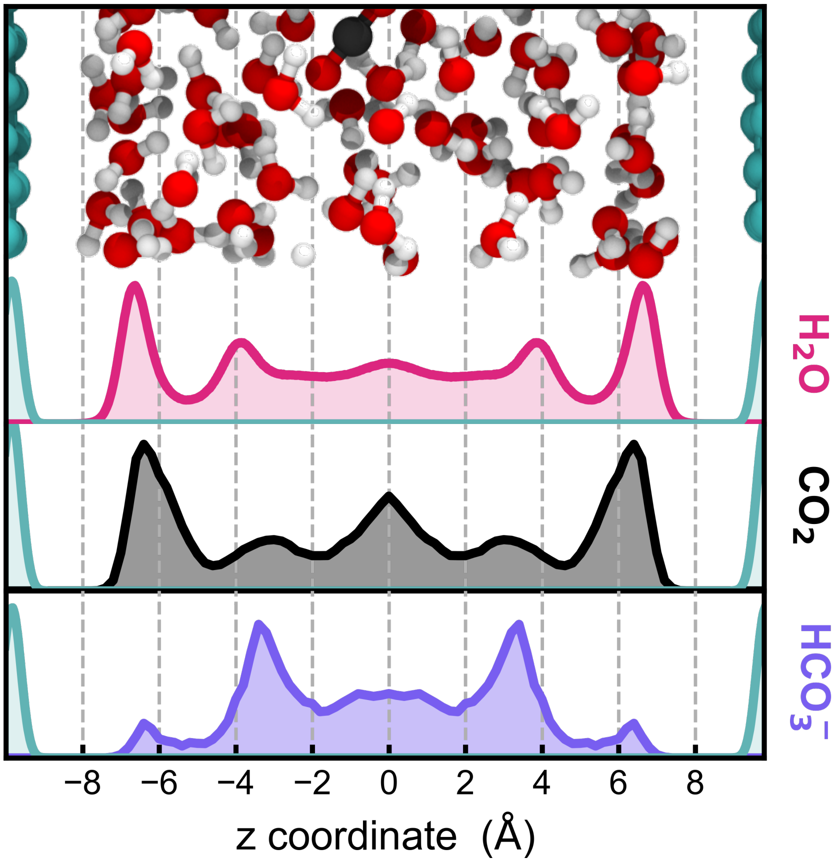

Figure S17: 20 Å pore density profiles obtained for water (pink), CO_2 (black), and HCO_3^- (purple), plotted as a function of the distance from the system center of mass. Profiles are shown alongside a snapshot of the overall system.

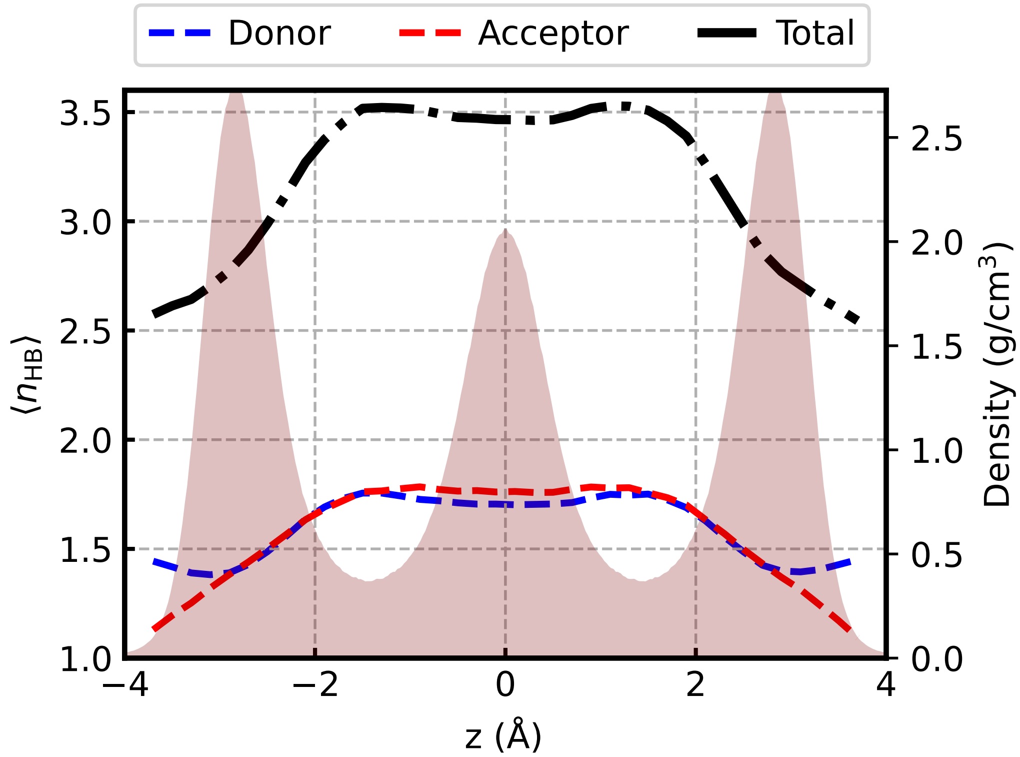

Figure S18: The nature of hydrogen bonding under pore-saturated conditions. The average hydrogen bond count, $\langle n_{\mathrm{HB}} \rangle$, is plotted as a function of the distance from the center of mass of the system (12 Å confinement). Hydrogen bonds are identified using a donor-acceptor distance of 3.5 Å and an O-H-O angle of 140°. The total hydrogen bond count (black) is decomposed into both donor (blue) and acceptor (red) contributions. The underlying water density (maroon) is plotted using the secondary axis.

References

(1) Liu, X.; Lyu, D.; Merlet, C.; Leesmith, M. J. A.; Hua, X.; Xu, Z.; Grey, C. P.; Forse, A. C. Structural Disorder Determines Capacitance in Nanoporous Carbons. *Science* **2024**, *384*, 321–325.

(2) Xu, Z.; Mapstone, G.; Coady, Z.; Wang, M.; Spreng, T. L.; Liu, X.; Molino, D.; Forse, A. C. Enhancing Electrochemical Carbon Dioxide Capture with Supercapacitors. *Nature Communications* **2024**, *15*, 7851.

(3) Binford, T. B.; Mapstone, G.; Temprano, I.; Forse, A. C. Enhancing the Capacity of Supercapacitive Swing Adsorption CO2 Capture by Tuning Charging Protocols. *Nanoscale* **2022**, *14*, 7980–7984.

(4) Forse, A. C.; Milner, P. J.; Lee, J.-H.; Redfearn, H. N.; Oktawiec, J.; Siegelman, R. L.; Martell, J. D.; Dinakar, B.; Zasada, L. B.; Gonzalez, M. I.; Neaton, J. B.; Long, J. R.; Reimer, J. A. Elucidating CO2 Chemisorption in Diamine-Appended Metal–Organic Frameworks. *Journal of the American Chemical Society* **2018**, *140*, 18016–18031.

(5) Forse, A. C.; Griffin, J. M.; Merlet, C.; Bayley, P. M.; Wang, H.; Simon, P.; Grey, C. P. NMR Study of Ion Dynamics and Charge Storage in Ionic Liquid Supercapacitors. *Journal of the American Chemical Society* **2015**, *137*, 7231–7242.

(6) Dickinson, L. M.; Harris, R. K.; Shaw, J. A.; Chinn, M.; Norman, P. R. Oxygen-17 and Deuterium NMR Investigation into the Adsorption of Water on Activated Carbon. *Magnetic Resonance in Chemistry* **2000**, *38*, 918–924.

(7) Hoffman, R. Solid-State Chemical-Shift Referencing with Adamantane. *Journal of Magnetic Resonance* **2022**, *340*, 107231.

(8) Morcombe, C. R.; Zilm, K. W. Chemical Shift Referencing in MAS Solid State NMR. *Journal of Magnetic Resonance* **2003**, *162*, 479–486.

(9) Harris, R. K.; Becker, E. D.; de Menezes, S. M. C.; Granger, P.; Hoffman, R. E.; Zilm, K. W. Further Conventions for NMR Shielding and Chemical Shifts (IUPAC Recommendations 2008). *Pure and Applied Chemistry* **2008**, *80*, 59–84.

(10) van Meerten, S. G. J.; Franssen, W. M. J.; Kentgens, A. P. M. ssNake: A Cross-Platform Open-Source NMR Data Processing and Fitting Application. *Journal of Magnetic Resonance* **2019**, *301*, 56–66.

(11) Bragg, R. J.; Griffiths, K.; Hwang, I.; Leketas, M.; Polus, K.; Presser, V.; Dryfe, R. A. W.; Griffin, J. M. Solvation Effects on Aqueous Ion Adsorption and Electrosorption in Carbon Micropores. *Carbon* **2024**, *229*, 119531.

(12) Lyu, D.; Märker, K.; Zhou, Y.; Zhao, E. W.; Gunnarsdóttir, A. B.; Niblett, S. P.; Forse, A. C.; Grey, C. P. Understanding Sorption of Aqueous Electrolytes in Porous Carbon by NMR Spectroscopy. *Journal of the American Chemical Society* **2024**, *146*, 9897–9910.

(13) Seravalli, J.; Ragsdale, S. W. ^{13}C NMR Characterization of an Exchange Reaction between CO and CO2 Catalyzed by Carbon Monoxide Dehydrogenase. *Biochemistry* **2008**, *47*, 6770–6781.

(14) Abbott, T. M.; Buchanan, G. W.; Kruus, P.; Lee, K. C. ^{13}C Nuclear Magnetic Resonance and Raman Investigations of Aqueous Carbon Dioxide Systems. *Canadian Journal of Chemistry* **1982**, *60*, 1000–1006.

(15) Vieira, R.; Marin-Montesinos, I.; Pereira, J.; Fonseca, R.; Ilkaeva, M.; Sardo, M.; Mafra, L. "Hidden" CO2 in Amine-Modified Porous Silicas Enables Full Quantitative NMR Identification of Physi- and Chemisorbed CO2 Species. *The Journal of Physical Chemistry C* **2021**, *125*, 14797–14806.

(16) Mafra, L.; Čendak, T.; Schneider, S.; Wiper, P. V.; Pires, J.; Gomes, J. R. B.; Pinto, M. L. Structure of Chemisorbed CO2 Species in Amine-Functionalized Meso-

porous Silicas Studied by Solid-State NMR and Computer Modeling. *Journal of the American Chemical Society* **2017**, *139*, 389–408.

(17) Omi, H.; Ueda, T.; Miyakubo, K.; Eguchi, T. Dynamics of CO2 Molecules Confined in the Micropores of Solids as Studied by ^{13}C NMR. *Applied Surface Science* **2005**, *252*, 660–667.

(18) Lahrar, E. H.; Merlet, C. Investigating the Effect of Particle Size Distribution and Complex Exchange Dynamics on NMR Spectra of Ions Diffusing in Disordered Porous Carbons through a Mesoscopic Model. *Faraday Discussions* **2024**,

(19) Perinu, C.; Arstad, B.; Jens, K.-J. ^{13}C NMR Experiments and Methods Used to Investigate Amine-CO2-H2O Systems. *Energy Procedia* **2013**, *37*, 7310–7317.

(20) Mani, F.; Peruzzini, M.; Stoppioni, P. CO2 Absorption by Aqueous NH3 Solutions: Speciation of Ammonium Carbamate, Bicarbonate and Carbonate by a ^{13}C NMR Study. *Green Chemistry* **2006**, *8*, 995–1000.

(21) Puziy, A. M.; Poddubnaya, O. I.; Socha, R. P.; Gurgul, J.; Wisniewski, M. XPS and NMR Studies of Phosphoric Acid Activated Carbons. *Carbon* **2008**, *46*, 2113–2123.

(22) Freitas, J. C. C.; Bonagamba, T. J.; Emmerich, F. G. Investigation of Biomass- and Polymer-Based Carbon Materials Using 13C High-Resolution Solid-State NMR. *Carbon* **2001**, *39*, 535–545.

(23) Freitas, J. C. C.; Emmerich, F. G.; Cernicchiaro, G. R. C.; Sampaio, L. C.; Bonagamba, T. J. Magnetic Susceptibility Effects on 13C MAS NMR Spectra of Carbon Materials and Graphite. *Solid State Nuclear Magnetic Resonance* **2001**, *20*, 61–73.

(24) Mincey, D. W.; Popovich, M. J.; Faustino, P. J.; Hurst, M. M.; Caruso, J. A. Monitoring of Electrochemical Reactions by Nuclear Magnetic Resonance Spectrometry. *Analytical Chemistry* **1990**, *62*, 1197–1200.

(25) Bhattacharyya, R.; Key, B.; Chen, H.; Best, A. S.; Hollenkamp, A. F.; Grey, C. P. In Situ NMR Observation of the Formation of Metallic Lithium Microstructures in Lithium Batteries. *Nature Materials* **2010**, *9*, 504–510.

(26) Ilott, A. J.; Chandrashekar, S.; Klöckner, A.; Chang, H. J.; Trease, N. M.; Grey, C. P.; Greengard, L.; Jerschow, A. Visualizing Skin Effects in Conductors with MRI: ^{7}Li MRI Experiments and Calculations. *Journal of Magnetic Resonance* **2014**, *245*, 143–149.

(27) He, S.; Morse, J. W. The Carbonic Acid System and Calcite Solubility in Aqueous Na-K-Ca-Mg-Cl-SO4 Solutions from 0 to 90°C. *Geochimica et Cosmochimica Acta* **1993**, *57*, 3533–3554.

(28) Lange, N. A.; Forker, G. M. *Handbook of Chemistry, a Reference Volume for All Requiring Ready Access to Chemical and Physical Data Used in Laboratory Work and Manufacturing*; New York, McGraw-Hill: New York (State), United States, 1961.

(29) Ishii, T.; Kashihara, S.; Hoshikawa, Y.; Ozaki, J.-i.; Kannari, N.; Takai, K.; Enoki, T.; Kyotani, T. A Quantitative Analysis of Carbon Edge Sites and an Estimation of Graphene Sheet Size in High-Temperature Treated, Non-Porous Carbons. *Carbon* **2014**, *80*, 135–145.

(30) Yoshii, T.; Nishikawa, G.; Prasad, V. K.; Shimizu, S.; Kawaguchi, R.; Tang, R.; Chida, K.; Sato, N.; Sakamoto, R.; Takatani, K.; Moreno-Rodríguez, D.; Škorňa, P.; Scholtzová, E.; Szilagyi, R. K.; Nishihara, H. Quantitative and Qualitative Analysis of Nitrogen Species in Carbon at the Ppm Level. *Chem* **2024**, *10*, 2450–2463.

(31) Yu, W. et al. Edge-Site-Free and Topological-Defect-Rich Carbon Cathode for High-Performance Lithium-Oxygen Batteries. *Advanced Science* **2023**, *10*, 2300268.

(32) Liu, H.; Pan, Z.-Z.; Aziz, A.; Tang, R.; Lv, W.; Nishihara, H. Nanoporous Membrane Electrodes with an Ordered Array of Hollow Giant Carbon Nanotubes. *Advanced Functional Materials* **2023**, *33*, 2303730.

(33) Batatia, I.; Kovacs, D. P.; Simm, G. N. C.; Ortner, C.; Csanyi, G. MACE: Higher Order Equivariant Message Passing Neural Networks for Fast and Accurate Force Fields. *Advances in Neural Information Processing Systems* **2022**, *35*, 11423–11436.

(34) Schran, C.; Thiemann, F. L.; Rowe, P.; Müller, E. A.; Marsalek, O.; Michaelides, A. Machine Learning Potentials for Complex Aqueous Systems Made Simple. *Proceedings of the National Academy of Sciences* **2021**, *118*, e2110077118.

(35) VandeVondele, J.; Krack, M.; Mohamed, F.; Parrinello, M.; Chassaing, T.; Hutter, J. Quickstep: Fast and Accurate Density Functional Calculations Using a Mixed Gaussian and Plane Waves Approach. *Computer Physics Communications* **2005**, *167*, 103–128.

(36) Kühne, T. D. et al. CP2K: An Electronic Structure and Molecular Dynamics Software Package - Quickstep: Efficient and Accurate Electronic Structure Calculations. *The Journal of Chemical Physics* **2020**, *152*, 194103.

(37) Perdew, J. P.; Burke, K.; Ernzerhof, M. Generalized Gradient Approximation Made Simple. *Physical Review Letters* **1996**, *77*, 3865–3868.

(38) Zhang, Y.; Yang, W. Comment on "Generalized Gradient Approximation Made Simple". *Physical Review Letters* **1998**, *80*, 890–890.

(39) Grimme, S.; Antony, J.; Ehrlich, S.; Krieg, H. A Consistent and Accurate Ab Initio Parametrization of Density Functional Dispersion Correction (DFT-D) for the 94 Elements H-Pu. *The Journal of Chemical Physics* **2010**, *132*, 154104.

(40) Bankura, A.; Karmakar, A.; Carnevale, V.; Chandra, A.; Klein, M. L. Structure, Dynamics, and Spectral Diffusion of Water from First-Principles Molecular Dynamics. *The Journal of Physical Chemistry C* **2014**, *118*, 29401–29411.

(41) Soper, A. K. The Radial Distribution Functions of Water and Ice from 220 to 673 K and at Pressures up to 400 MPa. *Chemical Physics* **2000**, *258*, 121–137.

(42) Skinner, L. B.; Huang, C.; Schlesinger, D.; Pettersson, L. G. M.; Nilsson, A.; Benmore, C. J. Benchmark Oxygen-Oxygen Pair-Distribution Function of Ambient Water from x-Ray Diffraction Measurements with a Wide Q-range. *The Journal of Chemical Physics* **2013**, *138*, 074506.

(43) Ohto, T.; Dodia, M.; Xu, J.; Imoto, S.; Tang, F.; Zysk, F.; Kühne, T. D.; Shigeta, Y.; Bonn, M.; Wu, X.; Nagata, Y. Accessing the Accuracy of Density Functional Theory through Structure and Dynamics of the Water–Air Interface. *The Journal of Physical Chemistry Letters* **2019**, *10*, 4914–4919.

(44) Plimpton, S. Fast Parallel Algorithms for Short-Range Molecular Dynamics. *Journal of Computational Physics* **1995**, *117*, 1–19.

(45) Thompson, A. P.; Aktulga, H. M.; Berger, R.; Bolintineanu, D. S.; Brown, W. M.; Crozier, P. S.; in 't Veld, P. J.; Kohlmeyer, A.; Moore, S. G.; Nguyen, T. D.; Shan, R.; Stevens, M. J.; Tranchida, J.; Trott, C.; Plimpton, S. J. LAMMPS - a Flexible Simulation Tool for Particle-Based Materials Modeling at the Atomic, Meso, and Continuum Scales. *Computer Physics Communications* **2022**, *271*, 108171.

(46) Kresse, G.; Hafner, J. Ab Initio Molecular Dynamics for Liquid Metals. *Physical Review B* **1993**, *47*, 558–561.

(47) Kresse, G.; Hafner, J. Ab Initio Molecular-Dynamics Simulation of the Liquid-Metal–Amorphous-Semiconductor Transition in Germanium. *Physical Review B* **1994**, *49*, 14251–14269.

(48) Kresse, G.; Furthmüller, J. Efficiency of Ab-Initio Total Energy Calculations for Metals and Semiconductors Using a Plane-Wave Basis Set. *Computational Materials Science* **1996**, *6*, 15–50.

(49) Kresse, G.; Furthmüller, J. Efficient Iterative Schemes for Ab Initio Total-Energy Calculations Using a Plane-Wave Basis Set. *Physical Review B* **1996**, *54*, 11169–11186.

(50) Kresse, G.; Joubert, D. From Ultrasoft Pseudopotentials to the Projector Augmented-Wave Method. *Physical Review B* **1999**, *59*, 1758–1775.

(51) Blöchl, P. E. Projector Augmented-Wave Method. *Physical Review B* **1994**, *50*, 17953–17979.

(52) Kästner, J.; Thiel, W. Bridging the Gap between Thermodynamic Integration and Umbrella Sampling Provides a Novel Analysis Method: "Umbrella Integration". *The Journal of Chemical Physics* **2005**, *123*, 144104.

(53) Afonso, R.; Gales, L.; Mendes, A. Kinetic Derivation of Common Isotherm Equations for Surface and Micropore Adsorption. *Adsorption* **2016**, *22*, 963–971.

(54) Skinner, L. B.; Benmore, C. J.; Neuefeind, J. C.; Parise, J. B. The Structure of Water around the Compressibility Minimum. *The Journal of Chemical Physics* **2014**, *141*, 214507.

(55) Daru, J.; Forbert, H.; Behler, J.; Marx, D. Coupled Cluster Molecular Dynamics of Condensed Phase Systems Enabled by Machine Learning Potentials: Liquid Water Benchmark. *Physical Review Letters* **2022**, *129*, 226001.
